# Molecular mechanisms underlying floral fragrance in *Camellia japonica* ‘High Fragrance’: a time-course assessment

**DOI:** 10.3389/fpls.2024.1461442

**Published:** 2024-11-12

**Authors:** Xuemei Chen, Xueping Zhang, Yongquan Li, Xueqin Tian, Xueyi Tian, Hongjie Zhao, Zuying Xuan, Kena Xue, Yongjuan Li, Wei Lin

**Affiliations:** ^1^ Foshan Institute of Forestry (Foshan Botanical Garden), Foshan, China; ^2^ College of Horticulture and Landscape Architecture, Zhongkai University of Agriculture and Engineering, Guangzhou, China

**Keywords:** *Camellia japonica* ‘High Fragrance’, floral scent, metabolome, transcriptome, terpenes

## Abstract

*Camellia japonica* ‘High Fragrance’ is a camellia hybrid known for its unique and intense floral scent. The current understanding of the dynamic changes in its fragrance and the underlying mechanisms are still limited. This study employed a combination of metabolomic and transcriptomic approaches to reveal the characteristics of the metabolites involved in the remarkable fragrance of this camellia and their biosynthetic mechanisms along three flower developmental stages (flower bud, initial bloom, and full bloom). Among the 349 detected volatile organic compounds (VOCs), the majority were terpenes (57, 16.33%) and esters (53, 15.19%). Of these, 136 VOCs exhibited differential accumulation over time. Transcriptomic data from floral organs at different flowering stages identified 56,303 genes, with 13,793 showing significant differential expression. KEGG enrichment analysis revealed 57, 91, and 33 candidate differential genes related to the biosynthesis of terpenes, phenylpropanoids, and fatty acid derivatives, respectively. This indicates that terpenes, esters, and their related synthetic genes might play a crucial role in the formation of ‘High Fragrance’ characteristics. During the entire flowering process, the majority of genes exhibited an elevated expression pattern, which correlated with the progressive accumulation of VOCs. Interestingly, the expression patterns of the differentially expressed genes in the mevalonate (MVA) and 2-C-methyl-D-erythritol 4-phosphate (MEP) pathways, associated with terpene synthesis, showed opposite trends. A transcriptional-metabolic regulatory network linking terpenoid compounds, related synthetic enzymes, and potential transcription factors could be outlined for ‘High Fragrance’ camellia, thus providing a theoretical basis for further exploring these events and breeding more fragrant camellias.

## Introduction

Floral scent, which includes the full spectrum of volatile metabolites emitted by flowers, is a key characteristic for evaluating the ornamental value of flowering plants (Knudsen et al., 2006), generally consisting of various low molecular weight volatile organic compounds (VOCs). Floral scents play crucial roles in attracting pollinators, deterring potential animal or insect pests, and facilitating inter-plant communication (Ramya et al., 2020). The composition of aromatic compounds varies due to factors including organ type, developmental stage, and environmental conditions (Li et al., 2020; Yang et al., 2022a). The biosynthesis of floral scent compounds is a complex process requiring the collaboration of multiple enzymes. These compounds are primarily classified into three groups: terpenoids, phenylpropanoids, and fatty acid derivatives, with terpenoids being the predominant volatiles (Roeder et al., 2007; Fu et al., 2016).

Terpenoids are synthesized via two separate pathways: the cytoplasmic mevalonate (MVA) pathway and the plastidial 2-C-methyl-D-erythritol 4-phosphate (MEP) pathway. These pathways lead to the production of isopentenyl diphosphate (IPP) and dimethylallyl diphosphate (DMAPP), the precursors of C5 compounds (Vranová et al., 2013). Terpene synthases (TPS) then catalyze the formation of terpenoids, which are further diversified by enzymatic cleavage (Rohmer, 2003; Yang et al., 2012). Phenylpropanoids are synthesized through the phenylalanine pathway (Dudareva and Pichersky, 2000), where phenylalanine ammonia-lyase (PAL) serves as the key enzyme converting phenylalanine to trans-cinnamic acid. Additional enzymes, including cinnamate 4-hydroxylase (C4H), 4-coumarate-CoA ligase (4CL), and chalcone synthase (CHS), further convert trans-cinnamic acid into various secondary metabolites (Ritter and Schulz, 2004; Zhang and Liu, 2015). The synthesis of fatty acid derivatives typically involves lipoxygenases (LOX) acting on linoleic and linolenic acids to produce hydroperoxides. These hydroperoxides are then cleaved by hydroperoxide lyase (HPL) to form aldehydes (Feussner and Wasternack, 2002). It is noteworthy that a considerable proportion of the products generated through the various synthetic pathways mentioned above are volatile compounds. Furthermore, the biosynthesis of volatile compounds is critically regulated by a range of transcription factors (TFs) (Jian et al., 2019; Ramya et al., 2019).

*Camellia japonica*, an evergreen plant from the Camellia genus, Theaceae family, is one of the world’s most prestigious ornamental plants, native to East Asia regions like China and Japan, and has been cultivated worldwide. Its flowers, diverse in color and form (Pereira et al., 2023a), contain metabolites such as proteins, polysaccharides, and polyphenols, offering medicinal and edible values (Pereira et al., 2023b). ‘High Fragrance’ is a hybrid camellia variety, bred from the red camellia ‘Mrs. Harms’ and other camellias. It features large, light pink, peony-shaped flowers with a long blooming period and a massive flower yield, possessing a unique and intense aroma, making it one of the most fragrant camellia varieties in the world (Fu et al., 2020). Due to the complexity and subtlety of floral scents, research on Camellia genus plant has primarily focused on flower shape and color (Fu et al., 2021; Fan et al., 2022). Studies on the floral scent of Camellia genus are relatively lacking. The first study employing GC-MS to investigate the aromatic components of various Camellia flowers revealed the presence of linalool and its oxides (Omata et al., 1989). Over the years, research on the floral scent of different members of Camellia genus has identified terpenoids as the main aromatic substances, with potential key components being ocimene, linalool, and geraniol (Jullien et al., 2008; Hattan et al., 2016). Fan et al. (2019a) found that 22 Camellia species released the highest concentration of volatile compounds during the semi-open stage, followed by a gradual decrease. These studies underscore the dynamism and diversity of volatile compounds in Camellia flowers, yet the molecular mechanisms underlying scent synthesis remain elusive.

As the market preference and breeding direction for flowers have shifted in recent years, there is a growing demand for fragrant camellias, yet aromatic varieties are rare. ‘High Fragrance’ known for its intense aroma and extended blooming period, serves as an ideal candidate for the study and breeding of fragrant camellias. However, the composition of its aromatic compounds and the regulatory mechanisms governing aroma formation are not yet fully understood. Understanding the molecular regulatory mechanisms of aromatic substance formation and accumulation in this plant is significantly hindered by the lack of transcriptomic and metabolomic data across different floral developmental stages. Advances in molecular biology techniques have made high-throughput sequencing a potent tool for exploring the biosynthesis mechanisms of volatile components in flowers. Headspace solid-phase microextraction (HS-SPME) combined with gas chromatography-mass spectrometry (GC-MS) is widely used for analyzing floral scents (Kutty et al., 2021; Zhang et al., 2021). Through transcriptome sequencing, the expression patterns of candidate genes involved in the biosynthesis of terpenoids and phenylpropanoids in the flowers of *Camellia sasanqua* and *Camellia sinensis* have been investigated within the genus Camellia (Huang et al., 2017; Mei et al., 2021). Previous researches have provided theoretical foundations for this study.

This research aims to uncover the dynamic properties and synthesis mechanisms of the ‘High Fragrance’ floral aroma. Using headspace solid-phase microextraction coupled with gas chromatography-mass spectrometry (HS-SPME-GC-MS), the study analyzed the spectrum of volatile components in ‘High Fragrance’ flowers across three developmental stages. Additionally, the study examined the genes’ expression patterns which were associated with fragrance using RNA sequencing (RNA-seq). By integrating data, the study identified key differential metabolites and related functional genes, unveiling changes in aromatic compounds throughout the ‘High Fragrance’ flower development stages. These findings deepen our understanding of the molecular synthesis and regulation of ‘High Fragrance’ floral aromas.

## Materials and methods

### Plant materials and experimental design

**Figure 1:**
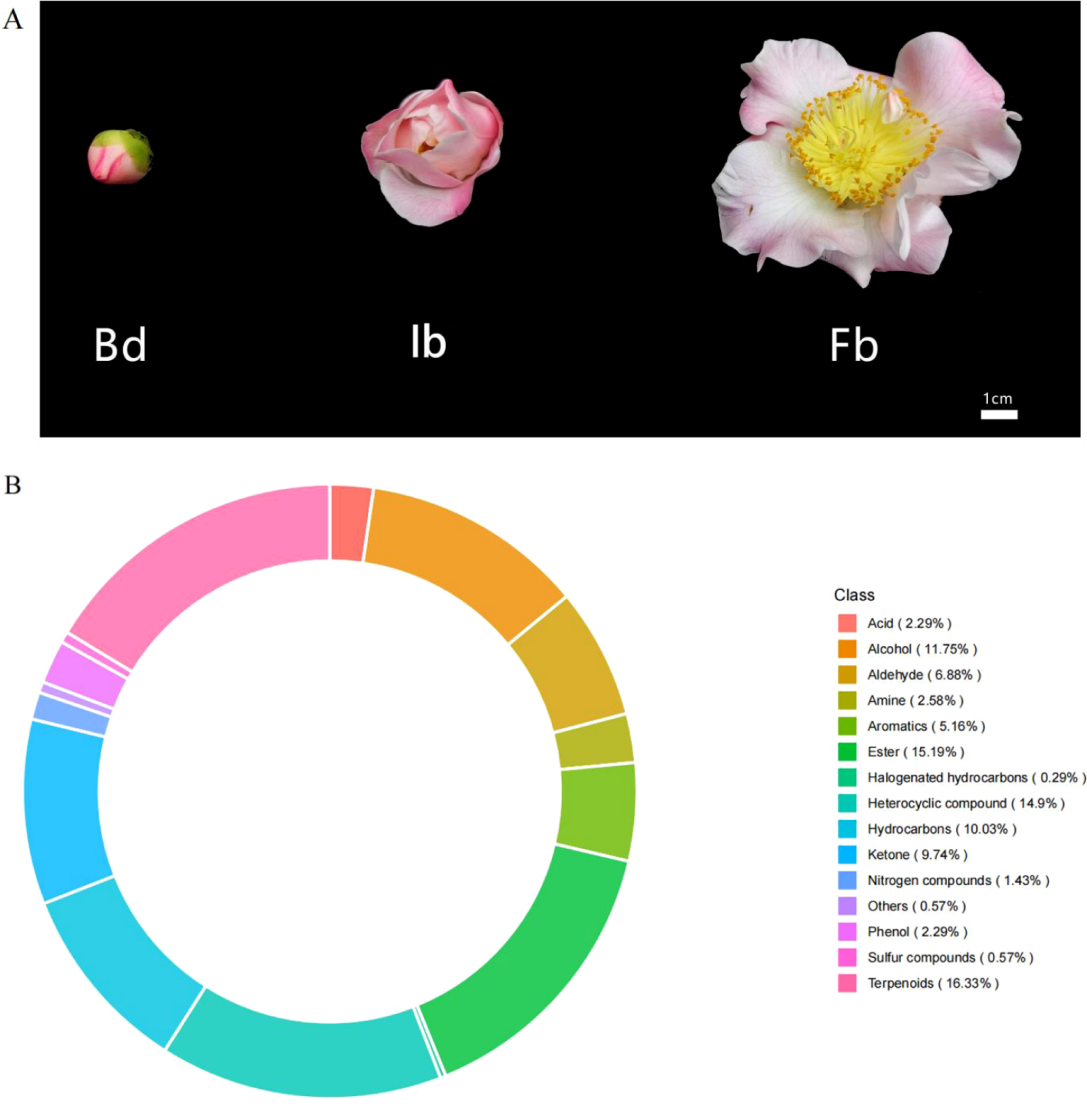
**(A)** Flowers of *C.japomica* ‘High Fragrance’ at different flowering stages. Bd, flower bud stage; Ib, initial bloom stage; Fb, full bloom stage; **(B)** Analysis of the types and proportions of volatile organic compounds in ‘High Fragrance’ flowers throughout all the three flowering stages.

The samples for this study were collected from 10 randomly selected ‘High Fragrance’ plants (15 years old) with similar growth, which are cultivated in the Camellia garden of Foshan Botanical Garden (Foshan, China). The region annual mean temperature is 21.9°C and annual mean precipitation is 1875.8 mm. To investigate the biosynthesis of floral scent components during flowering stages, whole flower were selected as the subject of study and collected at three key phases: flower bud (Bd), initial bloom (Ib), and full bloom (Fb) (**Figure 1A**). Sampling was strategically done between 10:00-11:00 AM on a clear day to reduce environmental variability. Samples were immediately cryopreserved in liquid nitrogen and maintained at -80°C after collection. For each flowering stage, seven biological replicates were prepared: three used for RNA sequencing and four used for volatile metabolomics analysis.

### RNA extraction

Total RNA from each sample was extracted using Trizol (Invitrogen, MA, USA) according to the manufacturer’s instructions. RNA degradation and contamination was monitored on 1% agarose gels and the integrity was assessed using the RNA Nano 6000 Assay Kit of the Bioanalyzer2100 system (Agilent Technologies, CA, USA). The Nano Photometer spectrophotometer (IMPLEN, CA, USA) was used to check RNA purity, and the Qubit^®^ 2.0 Fluorometer (Life Technologies, CA, USA) was used to measure the concentration.

### HS-SPME-GC-MS analysis

Samples were ground to powder in liquid nitrogen. 500 mg of the powder was transferred immediately to a 20 mL head-space vial containing NACL saturated solution, to inhibit any enzyme reaction. Volatiles were collected by headspace solid-phase microextraction (HS-SPME) using a 120 µm divinylbenzene/carbon/polydimethy-lsiloxane fiber (Agilent Technologies, CA, USA) at 100°C for 15 minutes. Volatiles were analyzed by gas chromatography-mass spectrometry (GC-MS) with an Agilent 8890 gas chromatograph and an Agilent 5977B mass spectrometer. The system was equipped with a 30 m × 0.25 mm × 0.25 µm DB-5MS capillary column (Agilent Technologies), and helium was used as the carrier gas at a flow rate of 1.2 mL/min. The injector temperature was set to 250°C. The oven temperature program was set as follows: start at 40°C for 3.5 minutes, increase to 100°C at 10°C/min, then to 180°C at 7°C/min, and finally to 280°C at 25°C/min, holding for 5 minutes. The quadrupole mass spectrometer, ion source, and transfer line were maintained at 150°C, 230°C, and 280°C, respectively. The mass spectrometer operated at 70 eV electron ionization potential, utilizing selected ion monitoring (SIM) mode for analyte identification and quantification. Volatile compounds were identified by matching their mass spectra with the MWGCSIM1 database. Using MassHunter (version B.08.00) to conduct the qualitative and quantitative analysis. Quantitative data normalization was performed using an internal standard. OPLS-DA analysis was used to derive Metabolite Variable Importance for Projection (VIP) values, which were then employed to identify differences in differential volatile organic compounds (DVOCs) across groups.

### RNA-seq and transcriptomic data analysis

Sequencing libraries were prepared using the NEBNext^®^ UltraTM RNA Library Prep Kit for Illumina^®^ (NEB, Ipswich, MA, USA) according to the manufacturer’s instructions. cDNA was generated using the AMPure XP system (Beckman Coulter, Beverly, MA, USA) and subsequently amplified by polymerase chain reaction (PCR). The product was validated for quality control using the Agilent Technologies 2100 Bioanalyzer and sequenced on the Illumina NovaSeq platform by MetWare Co. (Wuhan, China).

Fastp (version 0.23.2) was used to trim the adapters and low-quality sequences (N base content > 10% or Q ≤ 20 base content > 50%) (Chen et al., 2018). The clean data were mapped to the tea plant genome (Wei et al., 2018). HISAT2 (version 2.2.1) was utilized to construct the reference genome index and align clean reads to it (Kim et al., 2015). Stringtie (version 1.3.3) and itak (version 1.6) were employed to predict novel genes and transcription factors, respectively (Pertea et al., 2015; Zheng et al., 2016). Functional genes underwent annotation using 6 public databases: GO (Gene Ontology), KOG/COG (clusters of orthologous groups of proteins), Pfam (protein families), Swiss-prot (manually annotated and reviewed protein sequence database), KEGG (Kyoto Encyclopedia of Genes and Genomes) and Nr (NCBI non-redundant protein sequences). Gene expression levels were estimated by calculating fragments per kilobase of exon model per million mapped fragments (FPKM). Differential expression analysis between samples was performed using the DESeq2 (version 1.22.1) (Love et al., 2014), identifying differentially expressed genes (DEGs) based on |log_2_ fold change| ≥ 1 and a false discovery rate (FDR) ≤ 0.05 threshold. DEGs underwent GO and KEGG enrichment analyses by clusterProfiler (version 3.10.1). Expression heatmaps and venn diagrams were plotted using the Metware Cloud platform.

### Integrated analysis of transcriptomics and volatilomics

The FPKM values of all differentially expressed genes (DEGs) across the sample groups were used to perform K-means clustering analysis. To further explore the relationship between genes and metabolites, Pearson correlation coefficients were calculated between transcription factor expression levels and the contents of differential metabolites using R (version 4.3.2). The resulting correlation network was visualized by Cytoscape (version 3.9.1).

### Gene expression validation by qRT-PCR

To validate 12 aroma-related genes by qRT-PCR, first-strand cDNA synthesis was performed using total RNA extracted from the same samples used for RNA-seq, utilizing Hifair^®^ III 1st Strand cDNA Synthesis SuperMix (gDNA digester plus) (YEASEN, Shanghai, China). The qRT-PCR was conducted on FQD-96A (BIOER, Hangzhou, China) instrument. The *GAPDH* gene was used as the internal reference gene, and the relative expression levels were calculated using the 2^-ΔΔCt^ method (Livak and Schmittgen, 2001). Each analysis included three biological replicates. The primers used are listed in **Supplementary Table 1**. Figures and statistical analysis were presented by GraphPad Prism10 (version 10.3.0).

## Results

### Changes in volatile metabolites of ‘High Fragrance’

To investigate the characteristics of volatile compounds in ‘High Fragrance’ flowers, HS-SPME-GC-MS was used to analyze samples from three flowering stages (Bd, Ib, and Fb). Among the 349 identified VOCs, 343 were found across all sampling stages, while 6 were unique to the Fb samples. Additionally, 12 and 9 compounds were not detected in the Bd and Ib stages, respectively (**Supplementary Table 2**). The identified compounds were categorized into 15 distinct groups: terpenes, esters, heterocyclic compounds, hydrocarbons, ketones, alcohols, aldehydes, aromatic hydrocarbons, amines, phenols, acids, halogenated hydrocarbons, sulfur compounds, nitrogen compounds, and other compound. Among them, the most numerous were terpenes (57, 16.33%), followed by ester (53, 15.19%) and heterocyclic compounds (52, 14.9%). These three substances are considered the primary organic components of the fragrance in ‘High Fragrance’ (**Figure 1B**).

**Figure 2:**
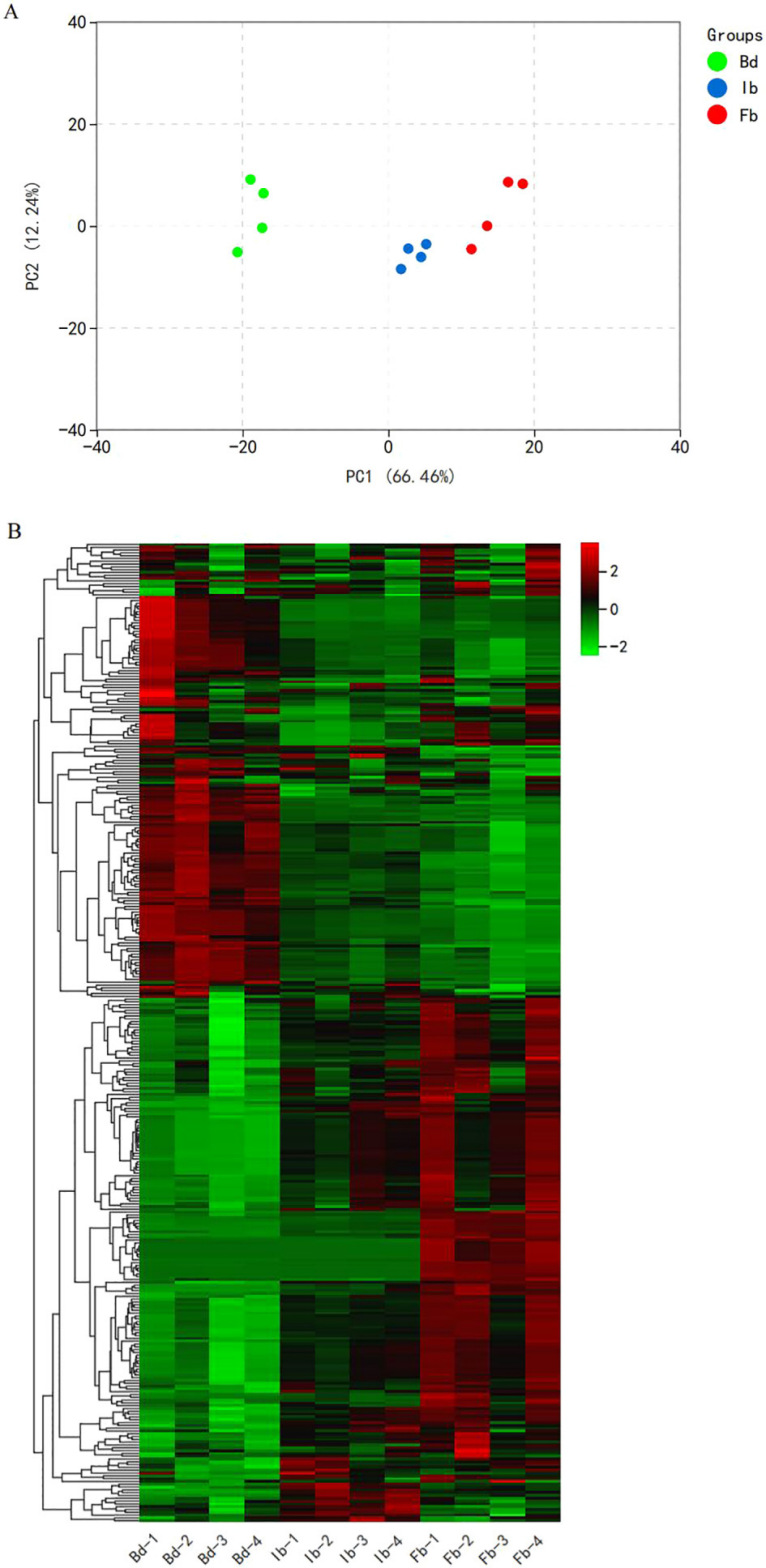
**(A)** Principal component analysis (PCA) of ‘High Fragrance’ flowers at different flowering stages by HS-SPME-GC/MS. **(B)** Hierarchical clustering heatmap of VOCs accumulation in ‘High Fragrance’ flowers.

Principal Component Analysis (PCA) results indicated that the first principal component (PC1) and the second principal component (PC2) accounted for 66.46% and 12.24% of the total variance, respectively (**Figure 2A**). Samples from three different flowering stages formed their own unique clusters, with significant differences observed between the Ib and Bd groups on PC1, while the Ib and Fb groups were more closely aligned. This indicates marked variations in the volatile metabolite profiles between the Bd and Ib stages. The positioning of the Ib group between the bud and full bloom stages was consistent with the sequential phases of floral development. Similarly, the hierarchical clustering heatmap revealed that the Ib and Fb groups clustered together before associating with the Bd group. The pattern of metabolite accumulation revealed two main clusters: one cluster increases gradually with flowering, while the other one shows opposite trend (**Figure 2B**). These differentially accumulated VOCs were crucial for elucidating mechanisms of aroma formation. The findings suggest that VOCs in ‘High Fragrance’ flower organs may accumulate and release rapidly before the initial bloom stage, and finally peaking at full bloom stage.

### Differential VOCs in different flower stages

**Figure 3:**
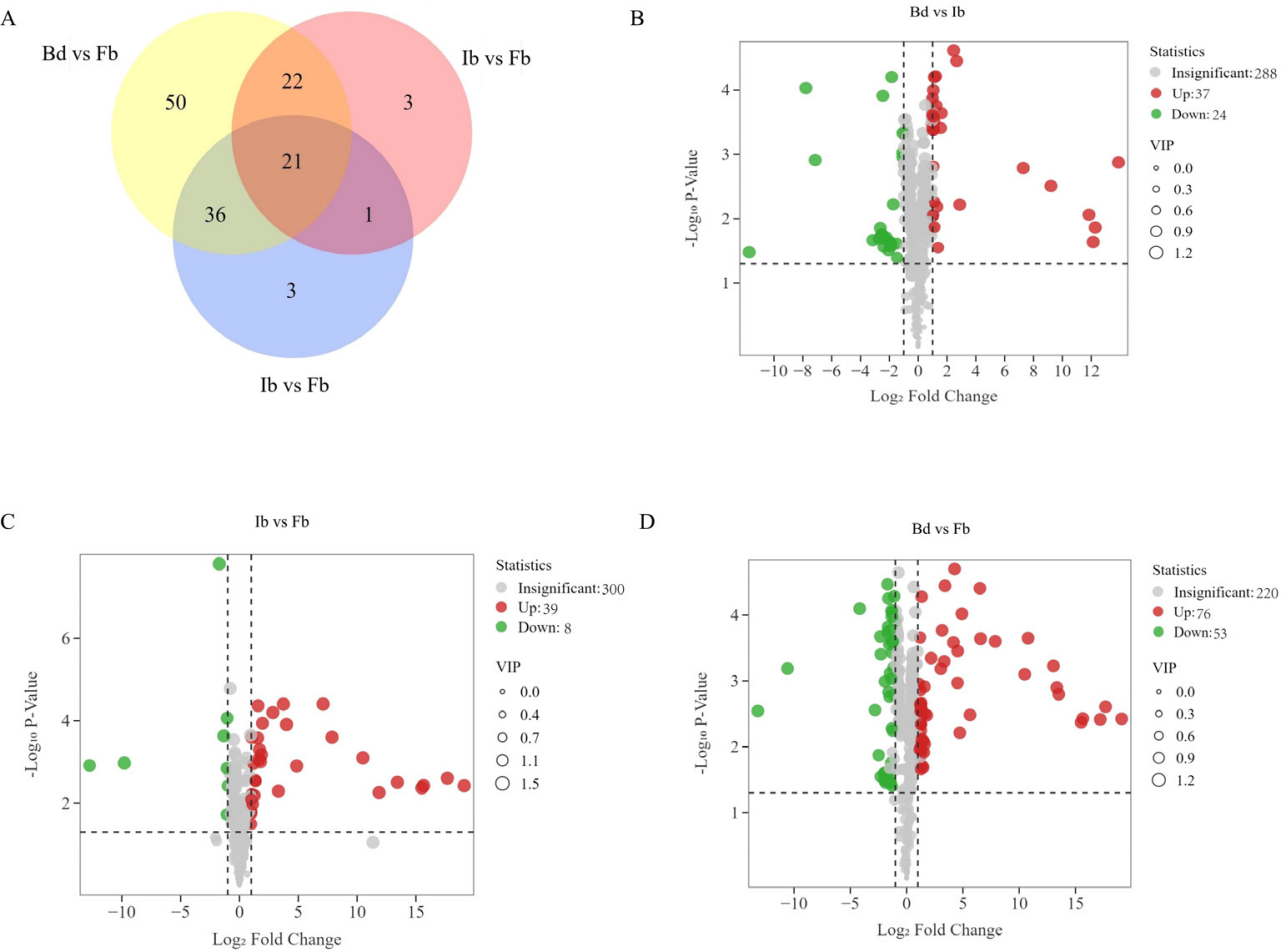
Differential volatile organic compounds (DVOCs) between different stages. **(A)** Venn diagram of DVOCs in ‘High Fragrance’ flowers. Volcano plots of DVOCs in **(B)** Bd vs Ib; **(C)** Ib vs Fb **(D)** Bd vs Fb.

Orthogonal partial least squares discriminant analysis (OPLS-DA) was employed to analyze the volatile metabolites across the three flower developmental stages, to further elucidate the differences in VOCs in ‘High Fragrance’ samples from various flowering stages. The R^2^X, R^2^Y, and Q^2^ values from the OPLS-DA model were all greater than 0.6, indicating the model’s strong discriminative ability (**Supplementary Figure 1**). The threshold of |Log_2_ fold change| ≥ 1.0 and a variable importance in the projection (VIP) value ≥ 1 were used to identify significantly different metabolites. We identified 136 unique differential metabolites across various stages: 61 between Bd and Ib, 47 between Ib and Fb, and 129 between Bd and Fb (**Figure 3A**). Among them, terpenoids were the most abundant (31, 22.79%), followed by esters (20, 14.71%), heterocyclic compounds (20, 14.71%), and alcohols (13, 9.56%), which is consistent with the overall volatile metabolites mentioned above. In the Bd vs Ib group, there were 37 upregulated and 24 downregulated compounds (**Figure 3B**); in the Ib vs Fb group, there were 39 upregulated and 8 downregulated VOCs (**Figure 3C**); and in the Bd vs Fb group, there were 76 upregulated and 53 downregulated VOCs (**Figure 3D**). These results once again prove that most VOCs accumulate gradually during flowering. Additionally, DVOCs flavor analysis showed that most green scent compounds decreased from Bd to Ib, and sweet scents compounds increased from Ib to Fb (**Supplementary Figure 2**).

**Figure 4:**
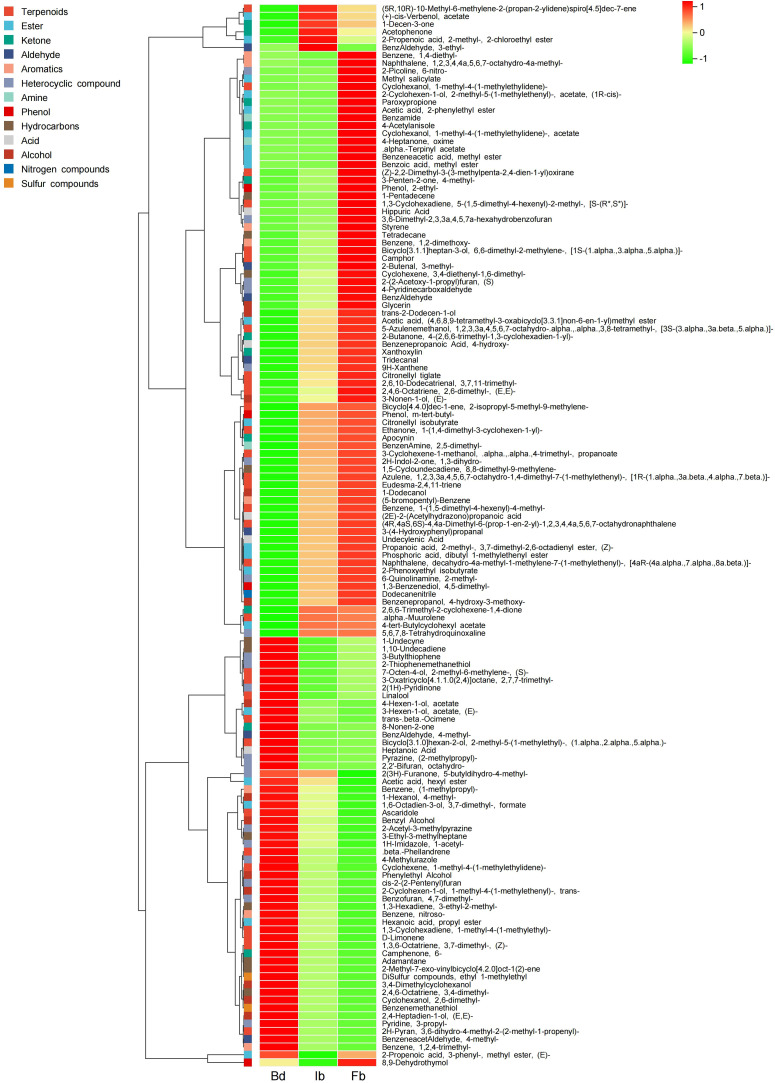
Hierarchical clustering heatmap of DVOCs at three stages of ‘High Fragrance’ flowers.

The DVOCs clustering heatmap shows that as the flowers gradually open, the 136 DVOCs can be roughly divided into two categories: the first category includes 98 compounds that accumulate gradually, with 15 VOCs not detected at the Bd stage but increasing as the flower opens. Among these, 13 VOCs showed significant upregulation in both the Bd vs Ib and Ib vs Fb comparisons, including camphor, 1,2-dimethoxy-Benzene, hippuric acid, (Z)-2,2-Dimethyl-3- (3-methylpenta-2,4-dien-1-yl)oxirane, [1S-(1.alpha.,3.alpha.,5.alpha.)]-6,6-dimethyl-2-methylene-bicyclo[3.1.1]heptan-3-ol, (S)-2-(2-Acetoxy-1-propyl)furan, 2-ethyl- Phenol, 4-methyl-benzaldehyde, 4-Pyridinecarboxaldehyde, glycerin, (E,E)-2,6-dimethyl-2,4,6-Octatriene, 3,4-diethenyl-1,6-dimethyl-cyclohexene, and (E)-3-Nonen-1-ol. Additionally, 6 DVOCs were only detected in the Fb samples, including Benzeneacetic acid methylester, benzamide, 4-acetylanisole, alpha.-Terpinyl acetate, 1-methyl-4-(1-methylethylidene)-cyclohexanol acetate, and 4-Heptanone oxime, contributing significantly to the fragrance of ‘High Fragrance’ at full bloom. Among them, 4-Acetylanisole, with a strong sweet scent, had a relatively high relative content and the largest fold change, potentially serving as a candidate biomarker for the aroma fingerprint of ‘High Fragrance’ at full bloom. The second category of 38 compounds showed the opposite pattern, with higher content in Bd samples and decreased content in Ib and Fb samples (**Figure 4**; **Supplementary Table 3**).

To investigate the variations in principal VOCs during the flowering stages of ‘High Fragrance’, we further screened 30 major volatile compounds with a relative content ≥1% in any flowering stage (**Supplementary Table 4**), with 16 major compounds decreasing in content as flowering progressed. Includes 7 alcohols, 5 esters, 4 terpenes, 4 aldehydes, 4 heterocyclic compounds, 3 ketones, 2 aromatic hydrocarbons, and 1 hydrocarbon. Among these, the levels of two compounds, Furan, 3-(4-methyl-3-pentenyl)- and (E)-2-hexenal, known for their strong woody and grassy scents, also decreased. Additionally, 13 VOCs significantly increased during the flowering period, including 1,2-dimethoxy-Benzene and methyl salicylate, which have intense sweet scent. In summary, during the development of ‘High Fragrance’ flower, key VOCs such as terpenes and esters dynamically change, giving ‘High Fragrance’ different aromas at different flowering stages.

### RNA-seq analysis

RNA sequencing (RNA-seq) of ‘High Fragrance’ flower samples at three developmental stages was conducted using the Illumina platform, resulting in the construction of 9 cDNA libraries. The Bd, Ib, and Fb samples retained 21.19 Gb, 21.97 Gb, and 21.38 Gb of clean reads, respectively. A total of 754,170,560 raw reads and 723,949,048 clean reads were obtained from the 9 samples. Among the clean reads, the average percentages of Q20 and Q30 bases were 97.56% and 93.14%, respectively. Additionally, 63.04% of clean reads uniquely aligned with the reference genome, with GC content ranging from 45.1% to 46.07%. The findings affirm the transcriptome data’s high quality, rendering it appropriate for subsequent analyses. A total of 56,303 unigenes were functionally annotated: 19,508 genes were annotated in all six databases, and 52,266 genes were annotated in at least one database.

**Figure 5:**
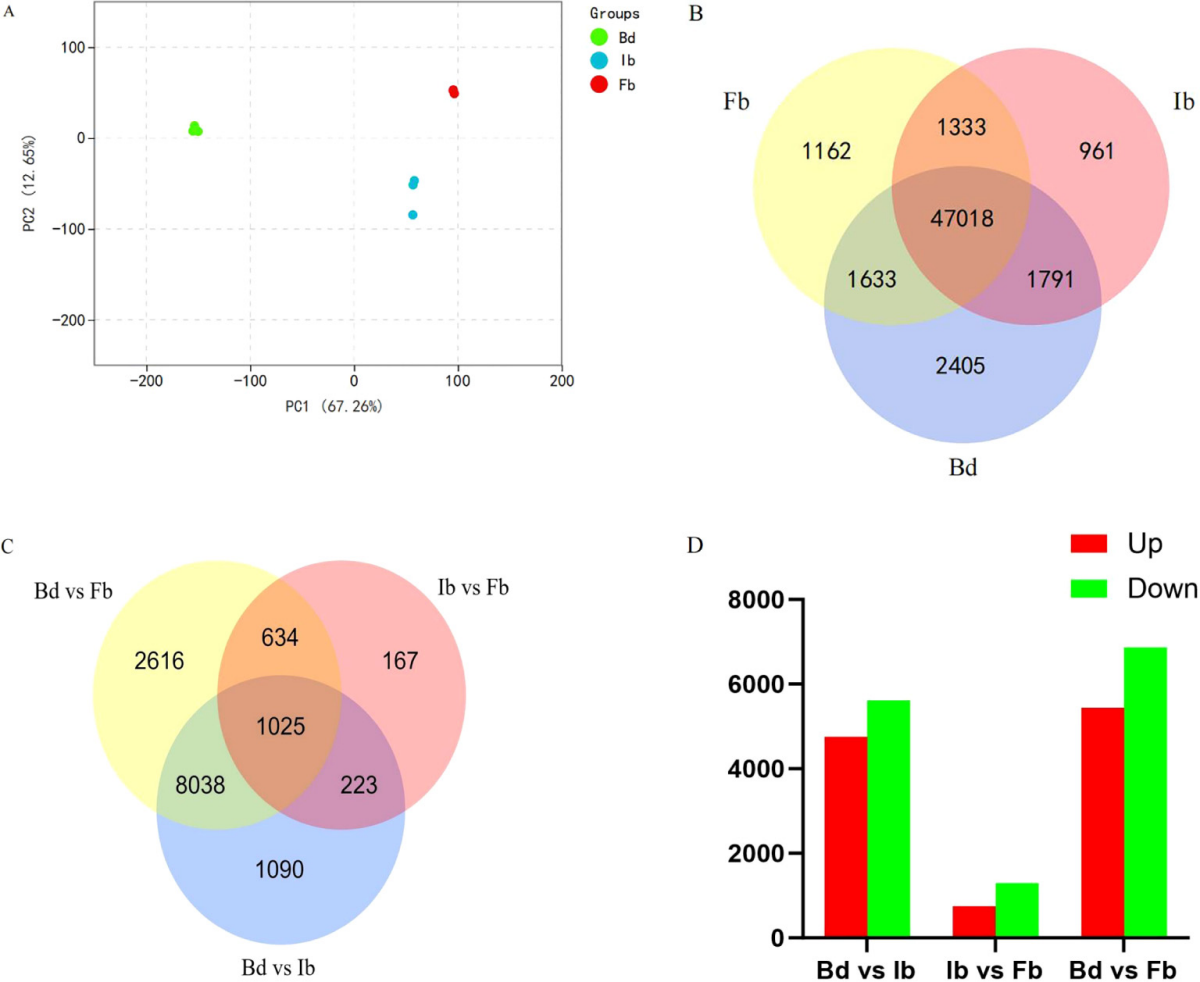
**(A)** Principal component analysis (PCA) of gene expression at various flowering stages. **(B)** Venn diagram of gene expression across three flower development stages. **(C)** Venn diagram of DEGs from three floral development stage comparisons. **(D)** Count of upregulated and downregulated genes in the three comparisons.

The Fragments Per Kilobase of exon model per Million mapped fragments (FPKM) method was used to quantify gene expression levels. PCA results indicated that the first principal component (PC1) and the second principal component (PC2) explained 67.26% and 12.65% of the total variance, respectively (**Figure 5A**). Three biological replicates of the three sample groups each clustered into one group, with Bd group significantly different from Ib and Fb group on PC1, while the gap between Ib and Fb was smaller, similar to the above VOCs results. We further screened for significantly differentially expressed genes between each comparison of flower samples, examining a total of 56,303 expressed genes. This included 52,847 genes in Bd samples, 51,103 in Ib samples, and 51,146 in Fb samples. Of these, 47,018 genes were consistently expressed across all three developmental stages, while 2,405, 961, and 1,162 genes were uniquely expressed at the Bd, Ib, and Fb stages, respectively (**Figure 5B**).

Using the following criteria to determine the significance of DEGs: |log_2_ fold change| ≥ 1 and adjusted p-value (FDR) < 0.05. Pairwise comparison analysis between these stage samples revealed a total of 13,793 DEGs (**Supplementary Table 5**). Among them, the Bd vs. Ib group had 10,376 DEGs, the Ib vs. Fb group had 2,049 DEGs, and the Bd vs. Fb group had 12,313 DEGs (**Figure 5C**). Compared to the Bd group, the Ib group had 4,759 genes upregulated and 5,617 genes downregulated. However, in the Ib vs Fb comparison, there were 750 upregulated and 1,299 downregulated DEGs (**Figure 5D**), indicating a gradual decrease in the number of DEGs during the ‘High Fragrance’ flowering process. This pattern suggests diminishing gene expression differences during the flowering period, aligning with the observed trends in VOC changes.

Using the FPKM based K-means clustering method to analyze the expression pattern of all the DEGs. The result illustrates that DEGs were categorized into four subclusters throughout the flowering stages, suggesting homogeneity in expression patterns and molecular functions within each subcluster (**Supplementary Figure 3**). The I and III subclusters showed an increase with flowering, the II subcluster continuously decreased, and the IV subcluster first increased then decreased. The II subcluster was the largest, containing 7,394 genes, whose expression levels continuously declined from the budding to the blooming stage. The I subcluster contained 2,314 genes with a pattern of continuous increase. The 3,011 genes in the III subcluster showed a trend of significant upregulation in the Ib stage followed by a plateau. The 1,074 genes in the IV subcluster exhibited a significant upregulation, peaking at the Ib stage, followed by a rapid decline.

### Differentially expressed genes in aroma-related synthesis pathway

KEGG enrichment analysis of the DEGs from each comparison was performed to categorize their functions and identify the metabolic pathways related to aroma. The results showed that 6 pathways might be related to the biosynthesis of floral scent, including “fatty acid biosynthesis” (ko00061), “linoleic acid metabolism” (ko00591), “terpenoid backbone biosynthesis” (ko00900), “sesquiterpenoid and triterpenoid biosynthesis” (ko00909), “phenylpropanoid biosynthesis” (ko00940), and “flavonoid biosynthesis” (ko00941) (**Supplementary Figure 4**; **Supplementary Table 6**). Given that terpenoids and esters are the main components of the ‘High Fragrance’ floral scent, particular attention was paid to the pathways related to these compounds. The linoleic acid metabolism pathway was consistently significantly enriched across all comparisons (p-value < 0.05). Additionally, in the comparison between the Bd and Ib stages, DEGs related to the terpenoid backbone biosynthesis pathway, as well as the sesquiterpenoid and triterpenoid biosynthesis pathways, showed notable enrichment. The enrichment factors were 0.14 and 0.22, with p-values of 0.02 and 1.78E-09, respectively. However, in the comparison between Ib and Fb stages, the enrichment factor and significance of these two pathways decreased. Conversely, the phenylpropanoid biosynthesis pathway, which was not significantly enriched in the Bd vs Ib comparison (rich factor: 0.16, p-value: 0.32), demonstrated significant increase in enrichment in the Ib vs Fb comparison (rich factor: 0.06, p-value: 8.77E-05). The fatty acid biosynthesis pathway was not significantly enriched in the Bd vs. Ib, and Ib vs. Fb, but shows a significant change in the Bd vs. Fb (p-value < 0.05), and this pathway produces precursors of esters, so this pathway also worth focus.

This suggests that as the flowering opening, the proportion of key DEGs on the terpenoid biosynthesis pathway decreases in overall expression, or the activity of these genes decreases. In contrast, the number of active differential expression genes in the phenylpropanoid and fatty acid biosynthesis pathway gradually increases, playing a more important role during the full bloom period. These results indicate that genes related to ko00900, ko00940, and ko00591 may play important roles in the biosynthesis of aromatic substances at different stages.

**Figure 6:**
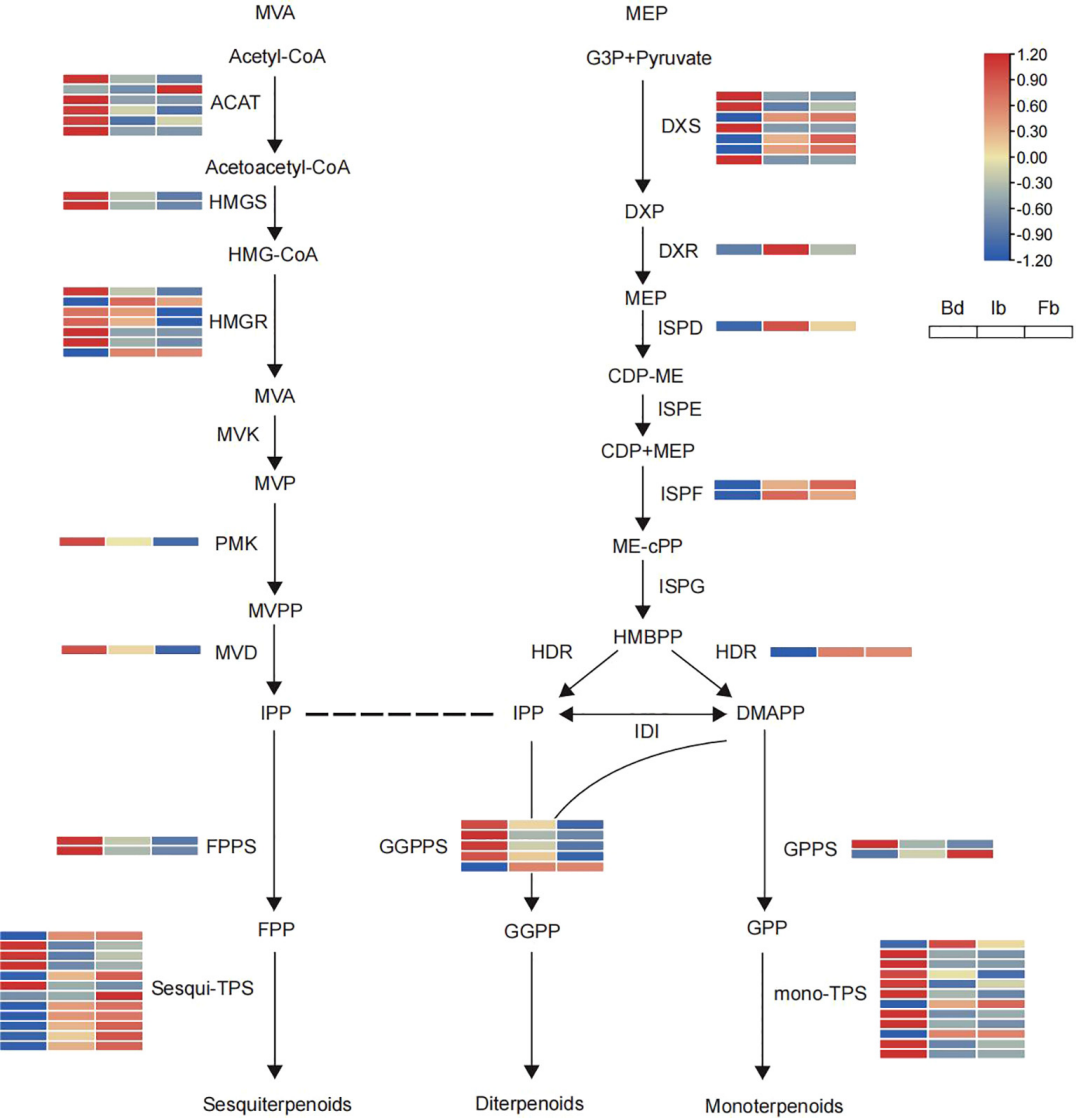
Heatmaps of the expression of terpenoid biosynthesis-related genes in ‘High Fragrance’ flowers at three developmental stages. The abbreviations of coding genes: ACAT, acetyl-CoA acetyltransferase; HMGS, HMG-CoA synthase; HMGR, HMG-CoA reductase; MVK, mevalonate kinase; PMK, phosphomevalonate kinase; MVD, diphosphomevalonate decarboxylase; DXS, 1-deoxy-dxylulose-5-phosphate synthase; DXR, 1-deoxy-d-xylulose-5-phosphate reductoisomerase; ISPD, 2-C-methyl-D-erythritol 4-phosphate cytidylyltransferase; ISPE, 4-diphosphocytidyl-2-C-methyl-D-erythritol kinase; ISPF, 2-C-methyl-D-erythritol 2,4-cyclodiphosphate synthase; ISPG, (E)-4-hydroxy-3-methylbut-2-enyl-diphosphate synthase; HDR, 4-hydroxy-3-methylbut-2-enyl diphosphate reductase; IDI, isopentenyl pyrophosphate isomerase; FPPS, farnesyl diphosphate synthase; GGPS, geranylgeranyl diphosphate synthase; GPS, geranyl diphosphate synthase; mono-TPS, monoterpenoid synthase; Sesqui-TPS, Sesquiterpenoid synthase.

The upstream biosynthetic pathways of terpenes include the MVA and MEP pathways, with 19 and 14 DEGs annotated, respectively (**Figure 6**). Notably, most of the differential genes in these two pathways showed opposite trends of change, with 14 genes in the MVA pathway, including *ACAT, HMGS, HMGR, PMK, MVD, FPPS*, showing high levels of transcription at the Bd stage and then gradually decreasing. In contrast, as flowering progressed, the MEP pathway showed an upregulation in the expression of 9 functional genes, including *DXS*, *DXR*, *ISPD*, *ISPF*, *HDR*, *GPPS*. Most genes belonging to these two pathways had continuous and stable expression during the flowering process. Terpene synthase (TPS), a pivotal enzyme for the diversity of terpenes, exhibited differential expression across 12 genes annotated to biosynthetic pathways associated with sesquiterpenes and monoterpenes. Notably, sesquiterpene synthase was predominantly expressed during the blooming phase, whereas monoterpene synthase demonstrated high expression levels during the bud stage.

Furthermore, the phenylpropanoid biosynthesis pathway was annotated with 91 DEGs encoding 14 known enzymes, with expression patterns varying across different flower developmental stages (**Supplementary Figure 5A**). Genes encoding key enzymes such as *PAL*, *4CL*, *COMT* showed higher transcriptional abundance in Fb compared to Bd or Ib. Meanwhile, genes such as *CCR, C4H*, and *CAD* exhibited a significant increase in expression in the Bd samples, yet their expression declined as the flowers progressively opened, evidenced by decreased expression abundance in both Ib and Fb samples. Additionally, the linoleic acid metabolism pathway, identified with 9 DEGs which encoding LOX, 7 of them were significantly upregulated at the bud stage. Similar gene expression trends were observed in the fatty acid biosynthesis pathway: 18 of 24 genes exhibited markedly higher expression levels at the Bd stage, followed by a decreasing trend (**Supplementary Figure 5B**).

### Combined analysis of metabolome and RNA-seq

**Figure 7:**
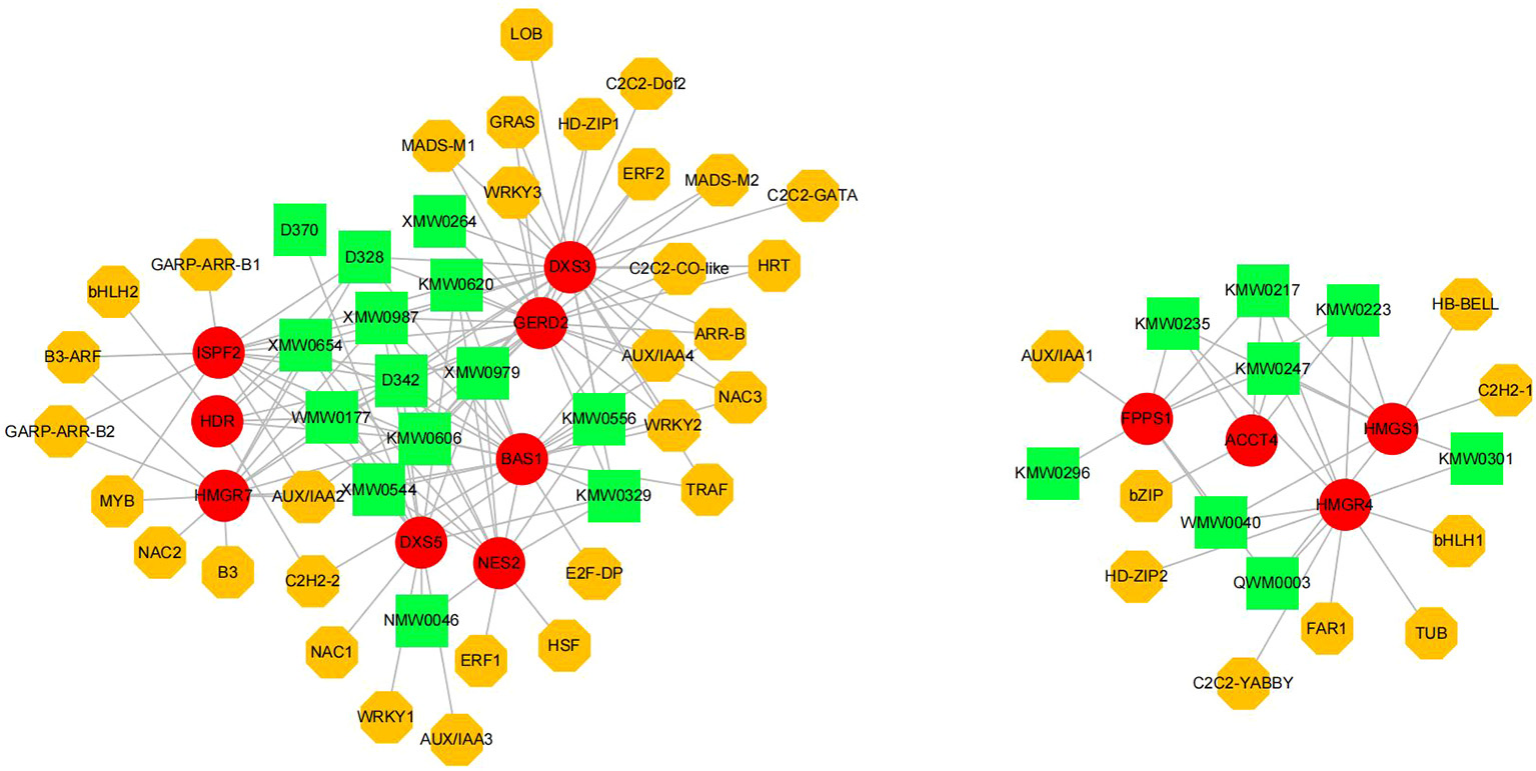
The regulatory network includes candidate transcription factors, terpenoids and terpene synthesis-related genes. Yellow octagons represented transcription factors, red circles represented structural genes, and green rectangle represented terpenoids. The gray line indicates a strong correlation (r > 0.9) between these elements.

Pearson correlation analysis was conducted to elucidate the relationships among terpenoid volatile metabolites, structural genes, and potential transcription factors (TFs), given the predominant role of terpenoid compounds in the aroma profile of ‘High Fragrance’. This analysis, focusing on DEGs related to terpenoid synthesis, all 57 terpenoid compounds, and 730 differential expressed TFs, applied a threshold of r > 0.9 and p < 0.05 to identify highly correlated DEGs, terpenoids, and TFs. Subsequently, we utilized Cytoscape to construct a network, exploring the regulatory dynamics within ‘High Fragrance’s terpenoid biosynthesis pathway. A significant number of DEGs were found to be involved in the terpenoid biosynthesis pathway, according to the analysis results. On the other hand, the transcription factors in the network include common TFs such as ERF, bHLH, NAC, and WRKY, suggesting they may play a regulatory role in the biosynthesis of terpene volatiles associated in ‘High Fragrance’. The network showed the correlation between 12 structural genes with 22 terpenoid metabolites and 43 TFs (**Figure 7**). The first part of the network includes 14 terpenes, 8 genes, and 31 TFs, and they all showed up-regulated trend during the flower opening process. DXS3, a crucial enzyme in the upstream MEP pathway of terpene synthesis, exhibits significant positive correlations with up to 12 aromatic terpenes, including Benzene-1-(1,5-dimethyl-4-hexenyl)-4-methyl-, Camphor, Naphthalene-decahydro-4a-methyl-1-methylene-7-(1-methylethenyl)-[4aR-(4a.alpha.,7.alpha.,8a.beta.)]-, Azulene, 1,2,3,3a,4,5,6,7-octahydro-1,4-dimethyl-7-(1-methylethenyl)-[1R-(1.alpha.,3a.beta.,4.alpha.,7.beta.)]-, 2,4,6-Octatriene-2,6-dimeth-yl-(E,E)-3-Cyclohexene-1-methanol, alpha,alpha-4-trimethyl-propanoate, alpha-Muu-rolene, Ethanone-1-(1,4-dimethyl-3-cyclohexen-1-yl)-, and 15 transcription factors, respectively. Among the downstream TPS, one GERD shows significant positive correlations with 14 transcription factors and 12 terpenes, respectively. Another part of the network includes 8 terpenes, 4 genes, and 9 transcription factors, whose expression patterns gradually decline during flowering. Four genes all involved in the MVA pathway. Except for the *ACCT, HMGR, HMGS*, and *FPPS* were positively correlated with 7 terpenes. Among them, *HMGR* was correlated with 5 TFs.

### Verification of representative genes expression by qRT-PCR

**Figure 8:**
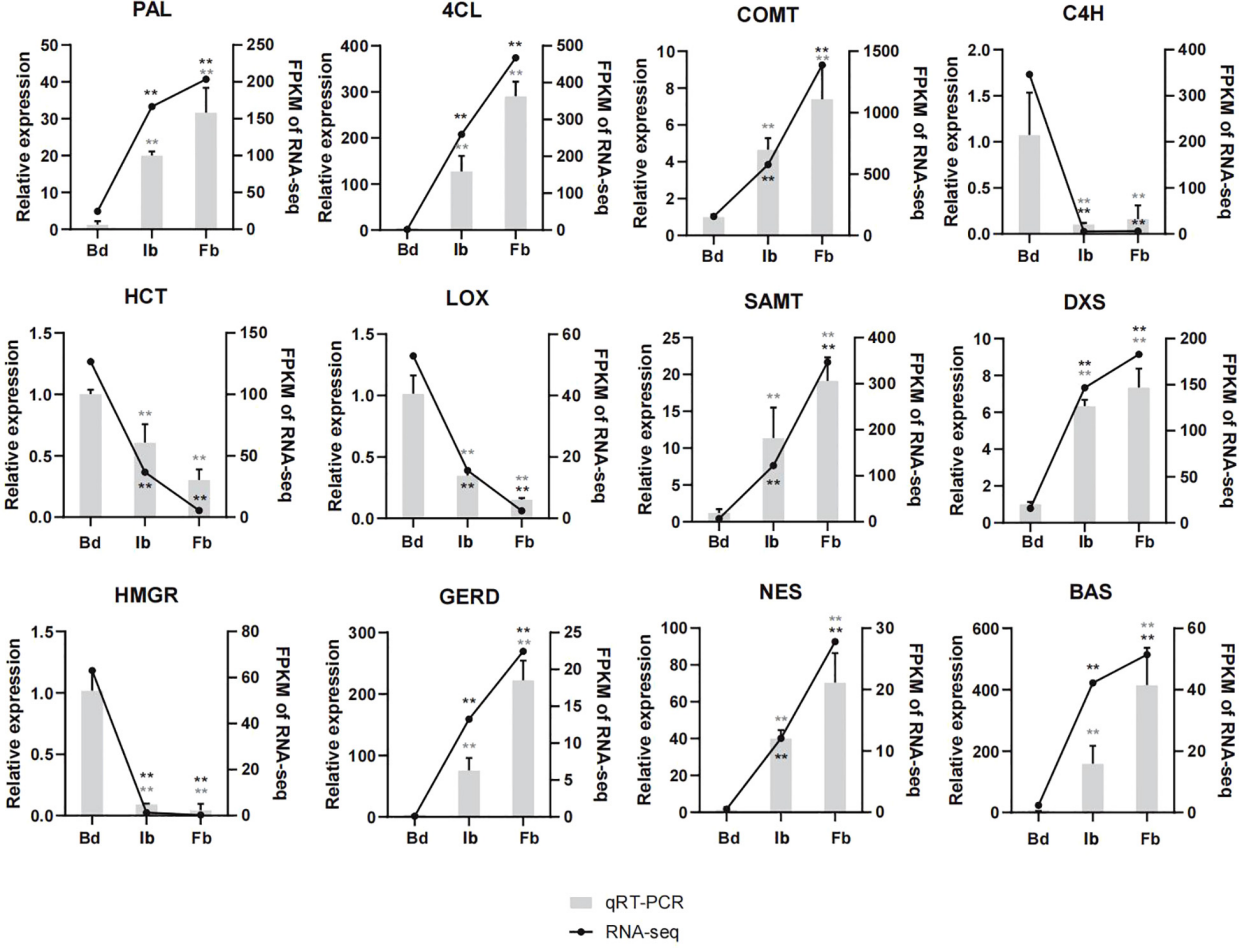
Expression patterns of 12 aroma-related genes by qRT-PCR verification assay. Two asterisks means p-value < 0.01. PAL, phenylalanine ammonia-lyase; 4CL, 4-coumarate-CoA ligase; COMT, caffeic acid Omethyltransferase; C4H, trans-cinnamate 4-monooxygenase; HCT, shikimate O-hydroxycinnamoyltransferase; LOX, linoleate 13S-lipoxygenase; SAMT, salicylic acid carboxyl methyltransferase; GERD, (-)-germacrene D synthase; NES, (3S,6E)-nerolidol synthase; BAS, beta-amyrin synthase. Other abbreviations were as shown in **Figure 6**.

For transcriptome data validation, we analyzed the expression of 12 genes involved in the biosynthesis of terpenes, phenylpropanoids, and fatty acids across Hb, Ib, and Fb samples by qRT-PCR. The RNA-seq results were confirmed by the largely consistent expression patterns of these genes, affirming the accuracy of the sequencing data (**Figure 8**).

## Discussion

### Flora scent profile of ‘High fragrance’

Floral scent, a key ornamental trait in flowering plants, often attracts consumers more effectively than color or other traits (Dudareva et al., 2013). Its composition exhibits significant variation across species and organs (Pichersky, 2023). Currently, *C.japonica* varieties with floral scent are very rare, and the intense fragrance of ‘High Fragrance’ is a key feature distinguishing it from other *C.japonica* flowers, including other naturally occurring wild species. In this study, employing HS-SPME-GC-MS, we identified 349 VOCs from the flower samples of ‘High Fragrance’ at three stages, significantly surpassing the quantity of volatile compounds reported in prior research. For instance, a study measuring the volatile compounds of mature ‘High Fragrance’ flowers with the same GC-MS method identified only 27 VOCs, with the most abundant compounds being aromatic and alcohol compounds (Yang et al., 2023a). This discrepancy could be due to differences in the method of flower sample collection, leading to the loss of volatile components.

Terpenoids and esters represent the most prevalent classes of volatile metabolites in plant floral scents, constituting the primary scent components in numerous aromatic plants (Song et al., 2018; Li et al., 2023). Present study found 187 volatile substances with different odors in the floral scent of ‘High Fragrance’, including 2-methoxy-3-isobutyl-pyrazine, 3-methyl-2-cyclohexen-1-one, 2-hexanoylfuran, Octanoic acid methyl ester, Isobornyl formate, and carvone, with a comparatively high relative content, which play a crucial role in the formation of floral scent. Terpenoids (57, 16.33%) and esters (53, 15.19%) are the most abundant components, similar to the volatile composition of genus camellia flower scent (Fan et al., 2019b). However, significant variations exist in the volatile composition of floral scents across species within the Camellia genus plants. For example, studies on *C.japonica* ‘Kramer’s supreme’ found that the most abundant volatiles in blooming flowers were alkanes and alcohols (Fan et al., 2005). In contrast, the most abundant volatiles in Camellia species like *C.sasanqua* ‘Dongmeigui’ and *C.saluenensis* Stapf ex Bean were alcohols and terpenoids (Qiu et al., 2015; Wang et al., 2018). Research indicates that linalool is the main constituent of the floral scent in most Camellia species, including *C.oleifera*, and *C.sinensis* (Gan et al., 2013). Although linalool and other substances were also detected in this study, their relative content was lower. In summary, the results of this study significantly enrich our understanding of the composition of camellia floral scent and provide many candidate substances for the future development of camellia fragrance products.

### Changes of VOCs emission in ‘High fragrance’ flower

During the flowering process, the release of floral scent is a dynamic process that changes continuously (Chen et al., 2010; Kim et al., 2022). The concentrations of VOCs in flowers are closely related to flower maturity, and plants begin to release floral scents when they are ready (Li et al., 2016; Fan et al., 2019a). This study observed that both the composition and emission levels of volatile compounds varied with flower development. Among the major differentiated compounds, 20 out of the most abundant 31 terpene substances show an increasing trend; likewise, 15 out of the 20 ester compounds also exhibit a significant accumulation trend. Specifically, the emission of most volatiles increased progressively from the bud to the full open stage, culminating in a peak at full bloom, indicative of a cumulative release process. This result is similar to many aromatic plants, for example, the scent substances of orchid flower cannot be perceived before reaching the fully bloomed stage (Zheng et al., 2023). Similarly, the synthesis and release of terpenoids and phenylpropanoids in *Rhododendron fortunei* also gradually increase as the flowers bloom (Yang et al., 2023b; Qin et al., 2024). The strongest phase of fragrance compound release in *Michelia alba* occurs during the full bloom period (Yong et al., 2023). However, in aromatic species like rose and *Murraya paniculata*, the peak of fragrance emission occurs during the initial opening stage (Oka et al., 2014; Paul et al., 2019), compared to which, ‘High Fragrance’ releases its fragrance substances over a longer period.

Floral volatiles play a significant role in biological interactions (Di Giusto et al., 2010; Muhlemann et al., 2014). Plants have evolved strategies to release specific floral scent components to better attract pollinators, while also possibly using certain compounds to defend against potential predators (Robacker et al., 1988). The floral scent complex may consist of both attractive and defensive components (Kessler et al., 2013). Scents are not only attractive to pollinators but can also affect the behavior and preferences of pollinators (Hambäck, 2016; Solís-Montero et al., 2018). In this study, among the 187 compounds with scents, 79 were significantly differentially expressed compounds, including 24 terpenes, 14 esters, 9 alcohols, 9 ketones, 8 heterocyclic compounds, 5 aldehydes, 3 aromatic hydrocarbons, 2 alkanes, 2 acids, 1 sulfur compound, 1 nitrogen compound, and 1 phenol. Forty-eight compounds gradually accumulated during the flowering process, including benzaldehyde, 1,2,2-dimethoxybenzene, methyl salicylate (MeSA), and p-anisyl acetone, which are relatively high in content and have a sweet aroma. The results of the differential compound flavor analysis also indicate that as ‘High Fragrance’ blooms, the green scent decreases while the sweet fragrance increases. Among these sweet compounds, MeSA has been demonstrated to not only effectively attract moths but also to serve as a robust defense against aphid and virus infections (Gong et al., 2023). These results suggest that ‘High Fragrance’ may effectively attract various potential pollinators and resist potential invading pathogens after the flowers are fully opened.

### Expression pattern of floral scent-related genes

Transcriptomic analysis has been widely used to identify genes involved in floral fragrance metabolism. The discovery of key genes in the volatile biosynthesis pathway has elucidated the molecular mechanisms regulating floral scent (Cai et al., 2016). The pathways of aromatic compound synthesis and enzyme activities regulate the biosynthesis and emission of floral fragrance (Dhandapani et al., 2017; Yue et al., 2023). There are some studies have been reported in camellia genus to identify genes involved in aroma synthesis (Gu et al., 2023). Despite this, the pathways related to floral scent synthesis in *C.japonica* still remain elusive. This study utilized RNA-seq analysis on ‘High Fragrance’ camellia floral organs across various developmental stages to methodically uncover gene expression patterns associated with scent biosynthesis, identifying 39,359 expressed genes, of which 13,793 were DEGs.

DEGs in the upstream pathway of terpene biosynthesis showed asynchrony. Nineteen and fourteen DEGs were detected in the MVA and MEP pathways, respectively. As the flowers opened, the number of downregulated DEGs in the MVA pathway was higher than the number of upregulated DEGs, while the opposite was true for the MEP pathway (MVA: 3 up/16 down; MEP: 9 up/5 down). Notably, 1 *ACAT*, 2 *HMGR*, 4 *DXS*, and 1 *GPPS* showed opposite trends to other genes in the MVA and MEP pathways, respectively. This implies that the homologous genes could exhibit diverse functions and expression profiles, resulting in the synthesis of varied compounds. Similarly, the expression trends of downstream TPS also showed asynchrony, with monoterpene synthase mainly expressed in buds, while sesquiterpene synthase was mainly expressed during the blooming period. This phenomenon of asynchrony, noted in both *C. indicum* var *aromaticum* and *D.chrysotoxum* (Du et al., 2022; Zhu et al., 2022), suggests a strong correlation between the diversity and synthesis of terpenoid compounds across various flower stages and the activation or suppression of specific DEGs. Additionally, differential expression of key enzymes related to phenylpropanoid, ester synthesis and accumulation was found in this study. The expression levels of 54 genes in the phenylpropanoid biosynthesis pathway increased as the flowers gradually opened, including 4 *PAL*, 4 *4CL*, and 3 *COMT* genes. In contrast, 37 DEGs showed decreased expression during the blooming period, including 4 *4CL*, 2 *C4H*, and 15 *CCR* genes. Unlike the synthesis pathways of terpenoids and phenylpropanoids, the enzymes closely associated with ester synthesis are mostly expressed at high abundance during the bud stage, like 7 of 9 *LOX* and 19 of 24 fatty acid biosynthesis related genes, followed by a gradual declined expression level. This trend is contrary to the accumulation trend of most ester compounds during the ‘High fragrance’ flowering process. We speculate that there might be a lag between the synthesis of esters and the expression of genes in ‘High fragrance’. The variation in gene expression highlights the evolutionary adaptation of these genes in producing a diverse array of products to meet ecological demands (Yang et al., 2022b). We hypothesize that in the aroma-related synthesis pathway, the distinct expression patterns of genes upstream and downstream contribute to the unique floral scent characteristics of ‘High Fragrance’ at various stages.

Overall, significant differential gene expression mostly occurs at or even before the initial bloom stage, with differential expression gradually decreasing as the flowers further open, consistent with the accumulation of metabolites. This suggests that most of the key biosynthetic regulation of ‘High Fragrance’ floral scent likely occurs before the initial opening phase, also explaining why some specific compounds are not detected in the buds. The identification of differentially expressed genes in the terpenoid and phenylpropanoid biosynthesis pathways offers new insights into the research on the floral scent of *C.japonica*.

### Transcriptional regulatory network for terpenoids biosynthesis

This research initially constructed a transcriptional regulatory network comprising transcription factors, terpenes, and terpene synthases, featuring 43 TFs, 22 terpenes, and 12 functional genes. Notably, many upstream MEP and MVA genes and downstream TPS are implicated in the biosynthesis of diverse terpenes. As key rate-limiting enzymes of the terpenoid biosynthesis pathway, the continuous and stable high expression of *DXS, HMGR*, and *HMGS* can provide sufficient precursors for the synthesis of downstream terpenes (Cordoba et al., 2011; He et al., 2021). In this study, *DXS3* showed a significant positive correlation with up to 14 aromatic terpenes and 12 TFs. Similarly, *HMGR7* was correlated with 7 terpenes and 6 TFs. These results imply that these two genes might be pivotal in their respective terpene synthesis pathways. Many TPSs are multi-product enzymes that can produce a variety of volatiles depending on the substrate, playing an important role in aromatic plants (Shimada et al., 2014; Pazouki and Niinemets, 2016). Notably, the *GRED2, BAS1*, and *NES2* in present regulatory network are highly correlated with the terpenes with scents in ‘High Fragrance’ flowers, including aromatic terpenes such as 2,4,6-Octatriene-2,6-dimethyl-(E,E)-, 3-Cyclohexene-1-methanol-alpha-alpha-4-tri-methyl-propanoate, alpha.-Muurolene, Ethanone, 1-(1,4-dimethyl-3-cyclohexen-1-yl)-, indicating they likely play key roles in the formation of ‘High Fragrance’ aroma.

This study identified 43 transcription factors from 28 families, such as the well-known bHLH, MYB, NAC, and WRKY, that show significant positive correlations with terpene synthesis genes. These transcription factors are known to regulate the production of volatile compounds in plant flowers. For example, *PbbHLH4* regulates monoterpenes synthesis in *Phalaenopsis bellina* (Chuang et al., 2018). In *Freesia hybrida*, two MYB transcription factors activate the expression of sesquiterpene synthase genes (Yang et al., 2020). In *Lilium* ‘Siberia’, *LiNAC100* plays a crucial role in monoterpene biosynthesis (Liu et al., 2024). In summary, both the functions of structural genes and the regulatory mechanisms of related TFs have significance and value for further exploration and warrant specific future research.

## Conclusion

Our study first report on transcriptome analysis of *C.japonica* ‘High Fragrance’, and combined metabolomic and transcriptomic analysis to elucidate the molecular mechanisms regulating volatile metabolite production in ‘High Fragrance’ flowers across three developmental stages. Specifically, terpenes and esters, along with other volatile metabolites, showed unique specificity patterns during various flower development stages, mirrored by numerous corresponding DEGs. Significant gene enrichment was notably observed in the biosynthesis pathways of terpenoids and phenylpropanoids. Members of the bHLH, ERF, NAC, and WRKY transcription factor families were identified as key regulators of ‘High Fragrance’ floral scent biosynthesis. This study’s findings may facilitate the identification of additional camellia floral scent-related genes, laying the groundwork for molecular breeding and metabolic engineering of aromatic camellias. Additionally, these insights may enhance the application of *C. japonica* floral scent products in consumer goods, medicinal products, and the industrial sector. This study can be conducted more precisely in future, for instance, by subdividing the flowering stages into more phases, thereby enabling better investigation of the dynamic patterns of changes in volatile metabolites and related genes.

## Data Availability

All data presented in this study are included in the article and **Supplementary Material**. Further inquiries can be directed to the corresponding author.

## Supplementary material

Supplementary Figure 1 The parameters from orthogonal partial least squares discriminant analysis (OPLS-DA) for each group indicate model stability and reliability, with values closer to 1 suggesting higher reliability. A model is considered valid with Q^2^> 0.5 and excellent with Q^2^ > 0.9. **(A)** Bd vs Ib; **(B**) Ib vs Fb; **(C)** Bd vs Fb.

Supplementary Figure 2 Flavor analysis of DVOCs of Bd vs Fb comparisons. Outer cycle list the name of different scents. The inner cycle list the name of the metabolites that have the correspondent scent.

Supplementary Figure 3 K-means cluster analysis of co-expressed gene and the corresponding expression patterns.

Supplementary Figure 4 Enrichment analysis of 6 candidate aroma-related pathway between different comparisons. **(A)** Bd vs Ib and **(B)** Bb vs Fb and **(C)** Id vs Fb. The rich factor is calculated by dividing the number of genes associated with the pathway among the DEGs by the number of genes associated with the pathway in the entire genome.

Supplementary Figure 5 Expression heatmaps of candidate genes involved in the biosynthesis of volatile metabolites in ‘High Fragrance’ flowers. **(A)** Phenylpropanoid biosynthesis pathway. **(B)** Fatty acid derivatives related genes.

## References

[B1] Cai J. Zu P. Schiestl F. P. (2016). The molecular bases of floral scent evolution under artificial selection: insights from a transcriptome analysis in Brassica rapa. Sci. Rep. 6, 36966. doi: 10.1038/srep36966 27841366 PMC5107913

[B2] Chen X. Kobayashi H. Suzuki Y. Sakai M. Shimeno T. Yamaguchi K.. (2010). Distribution and accumulation of floral scent compounds in rose flowers, and the change in the gene expression involved in the biosynthesis of these compounds during the unfurling process. Acta Hortic. 870, 235–240. doi: 10.17660/ActaHortic.2010.870.31

[B3] Chen S. Zhou Y. Chen Y. Gu J. (2018). fastp: an ultra-fast all-in-one FASTQ preprocessor. Bioinformatics 34, i884–i890. doi: 10.1093/bioinformatics/bty560 30423086 PMC6129281

[B4] Chuang Y.-C. Hung Y.-C. Tsai W.-C. Chen W.-H. Chen H.-H. (2018). PbbHLH4 regulates floral monoterpene biosynthesis in Phalaenopsis orchids. J. Exp. Bot. 69, 4363–4377. doi: 10.1093/jxb/ery246 29982590 PMC6093345

[B5] Cordoba E. Porta H. Arroyo A. San Román C. Medina L. Rodríguez-Concepción M.. (2011). Functional characterization of the three genes encoding 1-deoxy-D-xylulose 5-phosphate synthase in maize. J. Exp. Bot. 62, 2023–2038. doi: 10.1093/jxb/erq393 21199890

[B6] Dhandapani S. Jin J. Sridhar V. Sarojam R. Chua N.-H. Jang I.-C. (2017). Integrated metabolome and transcriptome analysis of Magnolia champaca identifies biosynthetic pathways for floral volatile organic compounds. BMC Genomics 18, 463. doi: 10.1186/s12864-017-3846-8 28615048 PMC5471912

[B7] Di Giusto B. Bessière J.-M. Guéroult M. Lim L. B. L. Marshall D. J. Hossaert-McKey M.. (2010). Flower-scent mimicry masks a deadly trap in the carnivorous plant Nepenthes rafflesiana. J. Ecol. 98, 845–856. doi: 10.1111/j.1365-2745.2010.01665.x

[B8] Du Z. Jin Y. Wang W. Xia K. Chen Z. (2022). Molecular and metabolic insights into floral scent biosynthesis during flowering in Dendrobium chrysotoxum. Front. Plant Sci. 13. doi: 10.3389/fpls.2022.1030492 PMC9742519 36518498

[B9] Dudareva N. Pichersky E. (2000). Biochemical and molecular genetic aspects of floral scents. Plant Physiol. 122, 627–633. doi: 10.1104/pp.122.3.627 10712525 PMC1539243

[B10] Dudareva N. Klempien A. Muhlemann J. K. Kaplan I. (2013). Biosynthesis, function and metabolic engineering of plant volatile organic compounds. New Phytol. 198, 16–32. doi: 10.1111/nph.12145 23383981

[B11] Fan Z. Li J. Li X. Yin H. (2019b). Composition analysis of floral scent within genus Camellia uncovers substantial interspecific variations. Scientia Hortic. 250, 207–213. doi: 10.1016/j.scienta.2019.02.050

[B12] Fan Z. Li J. Tian M. Li X. (2005). Analysis of aroma constituents of camellia variety Kramer’s supreme. Forestry Res. 18, 412–415. doi: 10.13275/j.cnki.lykxyj.2005.04.008

[B13] Fan M. Zhang Y. Yang M. Wu S. Yin H. Li J.. (2022). Transcriptomic and chemical analyses reveal the hub regulators of flower color variation from Camellia japonica bud sport. Horticulturae 8, 129. doi: 10.3390/horticulturae8020129

[B14] Fan J. Zhang W. Zhang D. Wang G. Cao F. (2019a). Flowering stage and daytime affect scent emission of Malus ioensis “Prairie rose. Molecules 24, 2356. doi: 10.3390/molecules24132356 31247958 PMC6650908

[B15] Feussner I. Wasternack C. (2002). The lipoxygenase pathway. Annu. Rev. Plant Biol. 53, 275–297. doi: 10.1146/annurev.arplant.53.100301.135248 12221977

[B16] Fu S. Chen B. Li Q. Wang Z. Yang S. Chen W.. (2020). An improved cultva camellia hybrid “high fragrance. Hubei Forestry Sci. Technol. 49, 82–83.

[B17] Fu J. Hou D. Wang Y. Zhang C. Bao Z. Zhao H. (2016). Floral scent composition and emission patterns of three symplocos species. J. Essential Oil Bearing Plants 19, 1873–1887. doi: 10.1080/0972060X.2016.1257369

[B18] Fu M. Yang X. Zheng J. Wang L. Yang X. Tu Y.. (2021). Unraveling the regulatory mechanism of color diversity in camellia japonica petals by integrative transcriptome and metabolome analysis. Front. Plant Sci. 12. doi: 10.3389/fpls.2021.685136 PMC8226227 34178004

[B19] Gan X. Liang Z. Wang D. Wang R. (2013). Analysis of aroma components in flowers of three kinds of camellia by HS-SPME/GC-MS. Food Sci. 34, 204–207.

[B20] Gong Q. Wang Y. He L. Huang F Zhang D Wang Y. (2023). Molecular basis of methyl-salicylate-mediated plant airborne defence. Nature 622, 139–148. doi: 10.1038/s41586-023-06533-3 37704724

[B21] Gu M. Gao T. Xu M. Hong Y. Wang Y. Yu J.. (2023). Identification and analysis of alleles in the aroma biosynthesis pathways based on Camellia sinensis ‘Jinguanyin’ haplotype-resolved genomes. Trees 37, 1627–1641. doi: 10.1007/s00468-023-02447-9

[B22] Hambäck P. A. (2016). Getting the smell of it–odour cues structure pollinator networks. J. Anim. Ecol. 85, 315–317. doi: 10.1111/1365-2656.12454 26899420

[B23] Hattan J. Shindo K. Ito T. Shibuya Y. Watanabe A. Tagaki C.. (2016). Identification of a novel hedycaryol synthase gene isolated from Camellia brevistyla flowers and floral scent of Camellia cultivars. Planta 243, 959–972. doi: 10.1007/s00425-015-2454-6 26744017

[B24] He Y. Chen H. Zhao J. Yang Y. Yang B. Feng L.. (2021). Transcriptome and metabolome analysis to reveal major genes of saikosaponin biosynthesis in Bupleurum chinense. BMC Genomics 22, 839. doi: 10.1186/s12864-021-08144-6 34798822 PMC8603497

[B25] Huang H. Xia E. H. Zhang H. Yao Q. Gao L. (2017). *De novo* transcriptome sequencing of Camellia sasanqua and the analysis of major candidate genes related to floral traits. Plant Physiol. Biochem. 120, 103–111. doi: 10.1016/j.plaphy.2017.08.028 28992542

[B26] Jian W. Cao H. Yuan S. Liu Y. Lu J. Lu W.. (2019). SlMYB75, an MYB-type transcription factor, promotes anthocyanin accumulation and enhances volatile aroma production in tomato fruits. Hortic. Res. 6, 22. doi: 10.1038/s41438-018-0098-y 30729012 PMC6355774

[B27] Jullien F. Gao J. Orel G. Legendre L. (2008). Analysis of tissue-specific emission of volatiles by the flowers of six *Camellia* species. Flavour Fragrance J. 23, 115–120. doi: 10.1002/ffj.1864

[B28] Kessler D. Diezel C. Clark D. G. Colquhoun T. A. Baldwin I. T. (2013). Petunia flowers solve the defence/apparency dilemma of pollinator attraction by deploying complex floral blends. Ecol. Lett. 16, 299–306. doi: 10.1111/ele.12038 23173705

[B29] Kim D. Langmead B. Salzberg S. L. (2015). HISAT: a fast spliced aligner with low memory requirements. Nat. Methods 12, 357–360. doi: 10.1038/nmeth.3317 25751142 PMC4655817

[B30] Kim H. Lee G. Song J. Kim S.-G. (2022). Real-time visualization of scent accumulation reveals the frequency of floral scent emissions. Front. Plant Sci. 13. doi: 10.3389/fpls.2022.835305 PMC9083826 35548271

[B31] Knudsen J. T. Eriksson R. Gershenzon J. Ståhl B. (2006). Diversity and distribution of floral scent. Bot. Rev. 72, 1. doi: 10.1663/0006-8101(2006)72[1:DADOFS]2.0.CO;2

[B32] Kutty N. N. Ghissing U. Mitra A. (2021). Revealing floral metabolite network in tuberose that underpins scent volatiles synthesis, storage and emission. Plant Mol. Biol. 106, 533–554. doi: 10.1007/s11103-021-01171-7 34263437

[B33] Li Y. Kong D. Fu Y. Sussman M. R. Wu H. (2020). The effect of developmental and environmental factors on secondary metabolites in medicinal plants. Plant Physiol. Biochem. 148, 80–89. doi: 10.1016/j.plaphy.2020.01.006 31951944

[B34] Li G. Liu J. Zhang H. Jia L. Liu Y. Li J.. (2023). Volatile metabolome and floral transcriptome analyses reveal the volatile components of strongly fragrant progeny of Malus × robusta. Front. Plant Sci. 14. doi: 10.3389/fpls.2023.1065219 PMC9895795 36743501

[B35] Li Y. Ma H. Wan Y. Li T. Liu X. Sun Z.. (2016). Volatile Organic Compounds Emissions from Luculia pinceana Flower and Its Changes at Different Stages of Flower Development. Molecules 21, 531. doi: 10.3390/molecules21040531 27110758 PMC6273779

[B36] Liu X. Yan W. Liu S. Wu J. Leng P. Hu Z. (2024). LiNAC100 contributes to linalool biosynthesis by directly regulating LiLiS in Lilium “Siberia. Planta 259, 73. doi: 10.1007/s00425-024-04340-2 38393405

[B37] Livak K. J. Schmittgen T. D. (2001). Analysis of relative gene expression data using real-time quantitative PCR and the 2(-Delta Delta C(T)) Method. Methods 25, 402–408. doi: 10.1006/meth.2001.1262 11846609

[B38] Love M. I. Huber W. Anders S. (2014). Moderated estimation of fold change and dispersion for RNA-seq data with DESeq2. Genome Biol. 15, 550. doi: 10.1186/s13059-014-0550-8 25516281 PMC4302049

[B39] Mei X. Wan S. Lin C. Zhou C. Hu L. Deng C.. (2021). Integration of metabolome and transcriptome reveals the relationship of benzenoid-phenylpropanoid pigment and aroma in purple tea flowers. Front. Plant Sci. 12. doi: 10.3389/fpls.2021.762330 PMC8649654 34887890

[B40] Muhlemann J. K. Klempien A. Dudareva N. (2014). Floral volatiles: from biosynthesis to function. Plant Cell Environ. 37, 1936–1949. doi: 10.1111/pce.12314 24588567

[B41] Oka N. Ohishi H. Hatano T. Hornberger M. Sakata K. Watanabe N. (2014). Aroma Evolution during Flower Opening in Rosa damascena Mill. Z. für Naturforschung C 54, 889–895. doi: 10.1515/znc-1999-1106

[B42] Omata A. Yomogida K. Nakamura S. Ota T. Izawa Y. (1989). Studies on the volatile compounds of camellia flowers. Engei Gakkai zasshi 58, 429–434. doi: 10.2503/jjshs.58.429

[B43] Paul I. Chatterjee A. Maiti S. Bhadoria P. B. S. Mitra A. (2019). Dynamic trajectories of volatile and non-volatile specialised metabolites in “overnight” fragrant flowers of Murraya paniculata. Plant Biol. (Stuttg) 21, 899–910. doi: 10.1111/plb.12983 30866144

[B44] Pazouki L. Niinemets Ü. (2016). Multi-substrate terpene synthases: their occurrence and physiological significance. Front. Plant Sci. 7 1019. doi: 10.3389/fpls.2016.01019 27462341 PMC4940680

[B45] Pereira A. G. Cassani L. Liu C. Li N. Chamorro F. Barreira J. C. M.. (2023a). Camellia japonica flowers as a source of nutritional and bioactive compounds. Foods 12, 2825. doi: 10.3390/foods12152825 37569093 PMC10417519

[B46] Pereira A. G. Cassani L. Oludemi T. Chamorro F. Calhelha R. C. Prieto M. A.. (2023b). Untargeted metabolomics and *in vitro* functional analysis unravel the intraspecific bioactive potential of flowers from underexplored Camellia japonica cultivars facing their industrial application. Ind. Crops Products 204, 117389. doi: 10.1016/j.indcrop.2023.117389

[B47] Pertea M. Pertea G. M. Antonescu C. M. Chang T.-C. Mendell J. T. Salzberg S. L. (2015). StringTie enables improved reconstruction of a transcriptome from RNA-seq reads. Nat. Biotechnol. 33, 290–295. doi: 10.1038/nbt.3122 25690850 PMC4643835

[B48] Pichersky E. (2023). Biochemistry and genetics of floral scent: a historical perspective. Plant J. 115, 18–36. doi: 10.1111/tpj.16220 36995899

[B49] Qin Y. Yang G. Li D. Zhang D. Chen Z. Yang Z.. (2024). Integrated analysis of the transcriptome and metabolome reveals genes involved in the synthesis of terpenoids in rhododendron fortunei lindl. Horticulturae 10, 959. doi: 10.3390/horticulturae10090959

[B50] Qiu J. Zhang Y. Chen J. Tian M. Xie Z. (2015). Study on the volatile components in flowers of 12 camellia species. Forestry Res. 28, 358–364. doi: 10.13275/j.cnki.lykxyj.2015.03.010

[B51] Ramya M. Jang S. An H.-R. Lee S.-Y. Park P.-M. Park P. H. (2020). Volatile organic compounds from orchids: from synthesis and function to gene regulation. Int. J. Mol. Sci. 21, 1160. doi: 10.3390/ijms21031160 32050562 PMC7037033

[B52] Ramya M. Park P. H. Chuang Y.-C. Kwon O. K. An H. R. Park P. M.. (2019). RNA sequencing analysis of Cymbidium goeringii identifies floral scent biosynthesis related genes. BMC Plant Biol. 19, 337. doi: 10.1186/s12870-019-1940-6 31375064 PMC6679452

[B53] Ritter H. Schulz G. E. (2004). Structural basis for the entrance into the phenylpropanoid metabolism catalyzed by phenylalanine ammonia-lyase. Plant Cell. 16, 3426-3436. doi: 10.1105/tpc.104.025288 15548745 PMC535883

[B54] Robacker D. C. Meeuse B. J. D. Erickson E. H. (1988). Floral Aroma: How far will plants go to attract pollinators? BioScience 38, 390–398. doi: 10.2307/1310925

[B55] Roeder S. Hartmann A.-M. Effmert U. Piechulla B. (2007). Regulation of simultaneous synthesis of floral scent terpenoids by the 1,8-cineole synthase of Nicotiana suaveolens. Plant Mol. Biol. 65, 107–124. doi: 10.1007/s11103-007-9202-7 17611797

[B56] Rohmer M. (2003). Mevalonate-independent methylerythritol phosphate pathway for isoprenoid biosynthesis. Elucidation and distribution. Pure Appl. Chem. 75, 375–388. doi: 10.1351/pac200375020375

[B57] Shimada T. Endo T. Fujii H. Rodríguez A. Peña L. Omura M. (2014). Characterization of three linalool synthase genes from Citrus unshiu Marc. and analysis of linalool-mediated resistance against Xanthomonas citri subsp. citri and Penicilium italicum in citrus leaves and fruits. Plant Sci. 229, 154–166. doi: 10.1016/j.plantsci.2014.09.008 25443842

[B58] Solís-Montero L. Cáceres-García S. Alavez-Rosas D. García-Crisóstomo J. F. Vega-Polanco M. Grajales-Conesa J.. (2018). Pollinator preferences for floral volatiles emitted by dimorphic anthers of a buzz-pollinated herb. J. Chem. Ecol. 44, 1058–1067. doi: 10.1007/s10886-018-1014-5 30191434

[B59] Song C. Wang Q. Teixeira da Silva J. yu X. (2018). Identification of floral fragrances and analysis of fragrance patterns in herbaceous peony cultivars. J. Am. Soc. Hortic. Sci. 143, 248–258. doi: 10.21273/JASHS04420-18

[B60] Vranová E. Coman D. Gruissem W. (2013). Network analysis of the MVA and MEP pathways for isoprenoid synthesis. Annu. Rev. Plant Biol. 64, 665–700. doi: 10.1146/annurev-arplant-050312-120116 23451776

[B61] Wang J. Li X. Yin H. Fan Z. Li J. (2018). Analysis on aroma components of Camellia sasanqua ‘Dongmeigui’at different flowering stages and floral organs. J. Plant Resources Environ. 27, 37–43. doi: 10.3969/J.issn.1674-7895.2018.01.05

[B62] Wei C. Yang H. Wang S. Zhao J. Liu C. Gao L.. (2018). Draft genome sequence of Camellia sinensis var. sinensis provides insights into the evolution of the tea genome and tea quality. Proc. Natl. Acad. Sci. U.S.A. 115, 201719622. doi: 10.1073/pnas.1719622115 PMC5939082 29678829

[B63] Yang D. Du X. Liang X. Han R. Liang Z. Liu Y.. (2012). Different roles of the mevalonate and methylerythritol phosphate pathways in cell growth and tanshinone production of Salvia miltiorrhiza hairy roots. PloS One 7, e46797. doi: 10.1371/journal.pone.0046797 23209548 PMC3510226

[B64] Yang Z. Li Y. Gao F. Jin W. Li S. Kimani S.. (2020). MYB21 interacts with MYC2 to control the expression of terpene synthase genes in flowers of Freesia hybrida and Arabidopsis thaliana. J. Exp. Bot. 71, 4140–4158. doi: 10.1093/jxb/eraa184 32275056

[B65] Yang Y.-Y. Ma B. Li Y.-Y. Han M.-Z. Wu J. Zhou X.-F.. (2022b). Transcriptome analysis identifies key gene LiMYB305 involved in monoterpene biosynthesis in Lilium “Siberia. Front. Plant Sci. 13. doi: 10.3389/fpls.2022.1021576 PMC9677127 36420028

[B66] Yang G. Qin Y. Jia Y. Xie X Li D Jiang B. (2023b). Transcriptomic and metabolomic data reveal key genes that are involved in the phenylpropanoid pathway and regulate the floral fragrance of Rhododendron fortunei. BMC Plant Biol. 23, 8. doi: 10.1186/s12870-022-04016-7 36600207 PMC9814181

[B67] Yang Y. Wang S. Leng P. Wu J. Hu Z. (2022a). Calcium and jasmonate signals mediate biosynthesis of the floral fragrance regulated by light quality in snapdragon. Plant Growth Regul. 97, 1–10. doi: 10.1007/s10725-022-00807-y

[B68] Yang M. Xie Y. Xu L. Xiang C. Fu S. Chen B-L. (2023a). Analysis of aroma volatiles in flowers of four camellia varieties. Hubei Forestry Sci. Technol. 62, 137–142+169. doi: 10.14088/j.cnki.issn0439-8114.2023.07.024

[B69] Yong Y. Yuan J. Jin X. Huang Y. Zhang Z. Chen Y.. (2023). Analysis of Aroma Volatiles from Michelia crassipes Flower and Its Changes in Different Flower Organs during Flowering. Horticulturae 9, 442. doi: 10.3390/horticulturae9040442

[B70] Yue Y. Wang L. Li M. Liu F. Yin J. Huang L.. (2023). A BAHD acyltransferase contributes to the biosynthesis of both ethyl benzoate and methyl benzoate in the flowers of Lilium oriental hybrid ‘Siberia.’. Front. Plant Sci. 14. doi: 10.3389/fpls.2023.1275960 PMC10570747 37841617

[B71] Zhang X. Liu C. J. (2015). Multifaceted regulations of gateway enzyme phenylalanine ammonia-lyase in the biosynthesis of phenylpropanoids. Mol Plant. 18, 17-27. doi: 10.1016/j.molp.2014.11.001 25578269

[B72] Zhang Y. Li C. Wang S. Yuan M. Li B. Niu L.. (2021). Transcriptome and volatile compounds profiling analyses provide insights into the molecular mechanism underlying the floral fragrance of tree peony. Ind. Crops Products 162, 113286. doi: 10.1016/j.indcrop.2021.113286

[B73] Zheng Y. Jiao C. Sun H. Rosli H. G. Pombo M. A. Zhang P.. (2016). iTAK: A program for genome-wide prediction and classification of plant transcription factors, transcriptional regulators, and protein kinases. Mol. Plant 9, 1667–1670. doi: 10.1016/j.molp.2016.09.014 27717919

[B74] Zheng B.-Q. Li X.-Q. Wang Y. (2023). New insights into the mechanism of spatiotemporal scent accumulation in orchid flowers. Plants (Basel) 12, 304. doi: 10.3390/plants12020304 36679016 PMC9866394

[B75] Zhu L. Liao J. Liu Y. Zhou C. Wang X. Hu Z.. (2022). Integrative metabolome and transcriptome analyses reveal the molecular mechanism underlying variation in floral scent during flower development of Chrysanthemum indicum var. aromaticum. Front. Plant Sci. 13. doi: 10.3389/fpls.2022.919151 PMC9889088 36733600

