# Supplementary figures and images for: Molecular mechanisms underlying floral fragrance in Camellia japonica ‘High Fragrance’: a time-course assessment
Source: Front Plant Sci. 2024 Nov 12;15:1461442. doi: 10.3389/fpls.2024.1461442 (PMC11588446; doi:10.3389/fpls.2024.1461442)

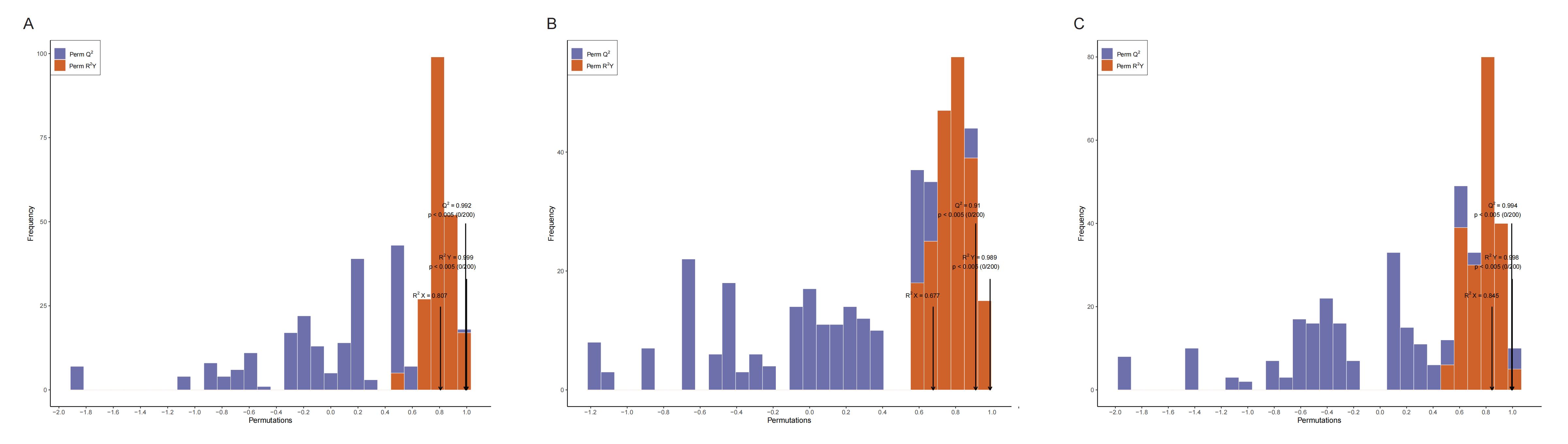

Supplement: Supplementary Figure 1 — The parameters from orthogonal partial least squares discriminant analysis (OPLS-DA) for each group indicate model stability and reliability, with values closer to 1 suggesting higher reliability. A model is considered valid with Q2> 0.5 and excellent with Q2 > 0.9. (A) Bd vs Ib; (B) Ib vs Fb; (C) Bd vs Fb. [file Image1.jpeg]

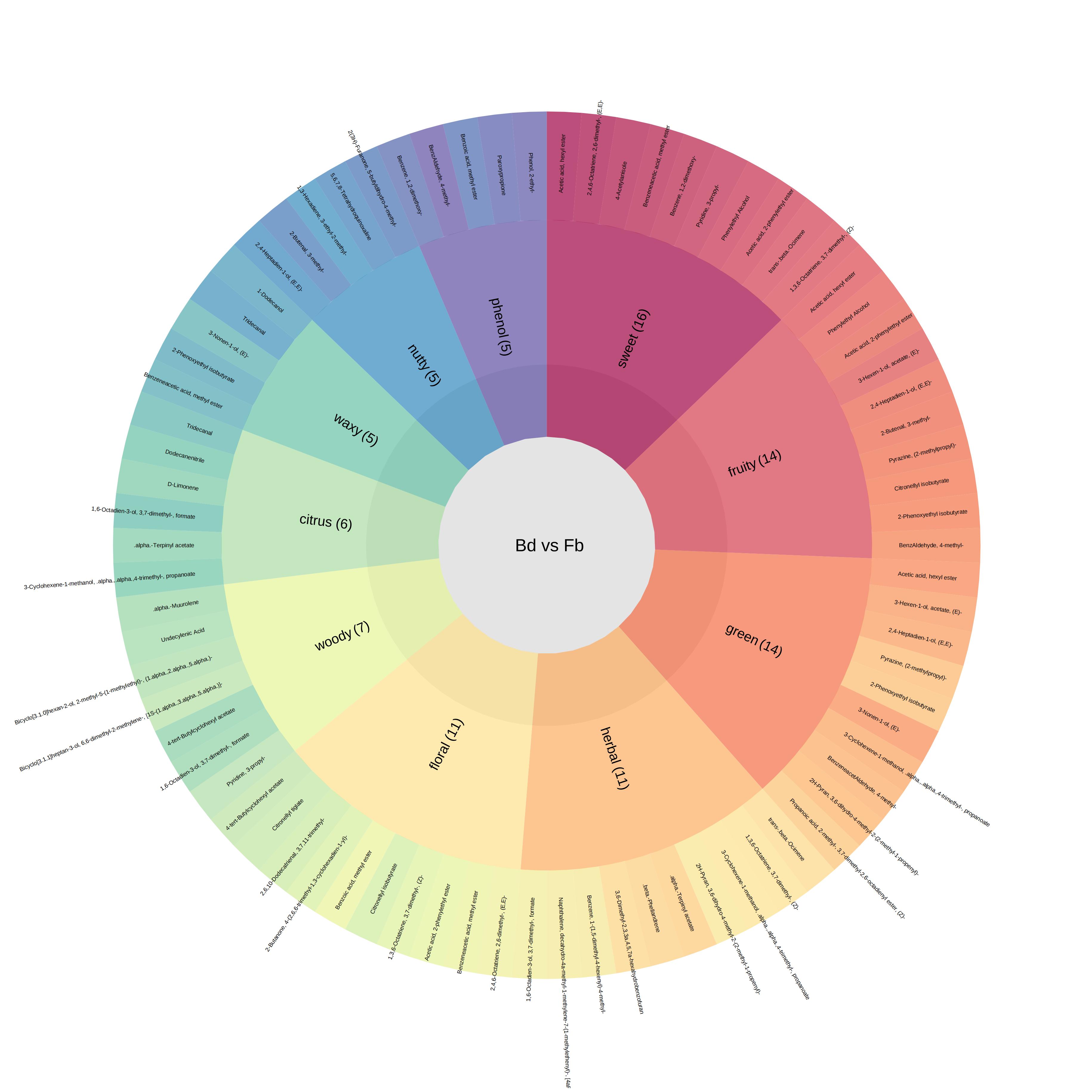

Supplement: Supplementary Figure 2 — Flavor analysis of DVOCs of Bd vs Fb comparisons. Outer cycle list the name of different scents. The inner cycle list the name of the metabolites that have the correspondent scent. [file Image2.jpeg]

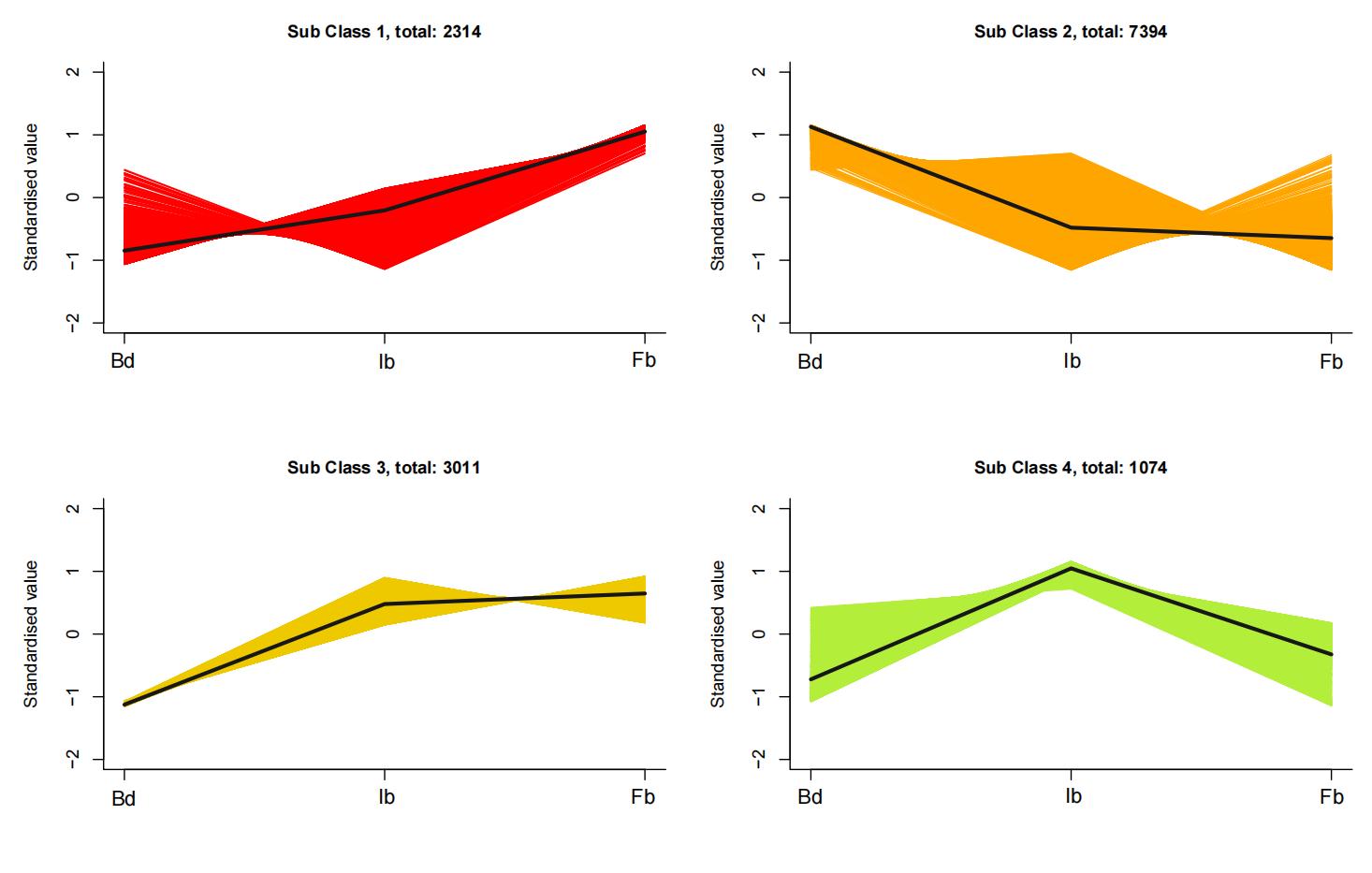

Supplement: Supplementary Figure 3 — K-means cluster analysis of co-expressed gene and the corresponding expression patterns. [file Image3.jpeg]

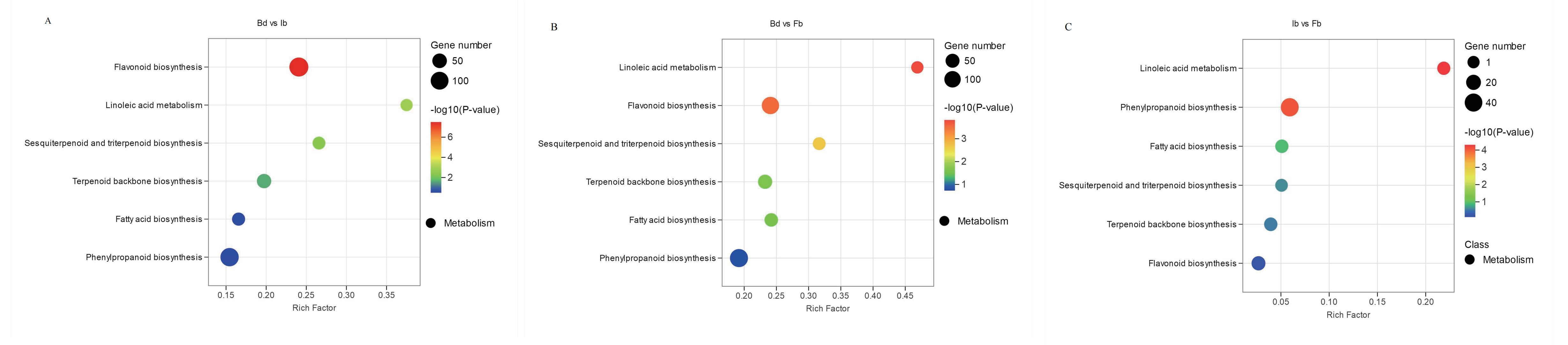

Supplement: Supplementary Figure 4 — Enrichment analysis of 6 candidate aroma-related pathway between different comparisons. (A) Bd vs Ib and (B) Bb vs Fb and (C) Id vs Fb. The rich factor is calculated by dividing the number of genes associated with the pathway among the DEGs by the number of genes associated with the pathway in the entire genome. [file Image4.jpeg]

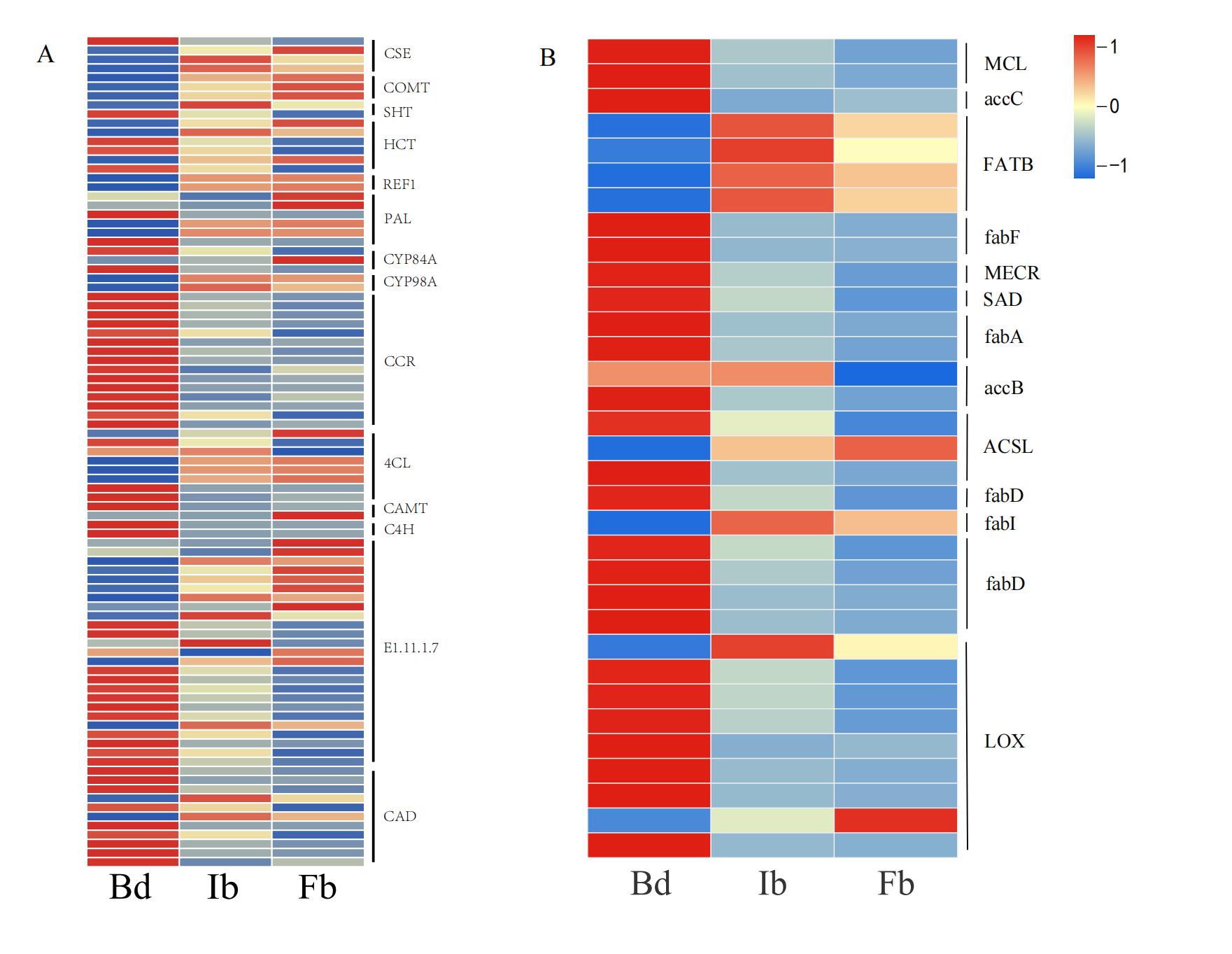

Supplement: Supplementary Figure 5 — Expression heatmaps of candidate genes involved in the biosynthesis of volatile metabolites in ‘High Fragrance’ flowers. (A) Phenylpropanoid biosynthesis pathway. (B) Fatty acid derivatives related genes. [file Image5.jpeg]
